# Phenotypic and transcriptional characterization of *F. tularensis* LVS during transition into a viable but non-culturable state

**DOI:** 10.3389/fmicb.2024.1347488

**Published:** 2024-02-06

**Authors:** Stuart Cantlay, Nicole L. Garrison, Rachelle Patterson, Kassey Wagner, Zoei Kirk, Jun Fan, Donald A. Primerano, Mara L. G. Sullivan, Jonathan M. Franks, Donna B. Stolz, Joseph Horzempa

**Affiliations:** ^1^Department of Biomedical Sciences, West Liberty University, West Liberty, WV, United States; ^2^Department of Biomedical Sciences, Joan C. Edwards School of Medicine, Marshall University, Huntington, WV, United States; ^3^Department of Cell Biology, Center for Biologic Imaging, University of Pittsburgh, Pittsburgh, PA, United States

**Keywords:** *Francisella tularensis*, viable but non-culturable (VBNC), RNA-Seq, transcriptomics, bacterial physiology, host-microbe interaction

## Abstract

*Francisella tularensis* is a gram-negative, intracellular pathogen which can cause serious, potentially fatal, illness in humans. Species of *F. tularensis* are found across the Northern Hemisphere and can infect a broad range of host species, including humans. Factors affecting the persistence of *F. tularensis* in the environment and its epidemiology are not well understood, however, the ability of *F. tularensis* to enter a viable but non-culturable state (VBNC) may be important. A broad range of bacteria, including many pathogens, have been observed to enter the VBNC state in response to stressful environmental conditions, such as nutrient limitation, osmotic or oxidative stress or low temperature. To investigate the transition into the VBNC state for *F. tularensis*, we analyzed the attenuated live vaccine strain, *F. tularensis* LVS grown under standard laboratory conditions. We found that *F. tularensis* LVS rapidly and spontaneously enters a VBNC state in broth culture at 37°C and that this transition coincides with morphological differentiation of the cells. The VBNC bacteria retained an ability to interact with both murine macrophages and human erythrocytes in *in vitro* assays and were insensitive to treatment with gentamicin. Finally, we present the first transcriptomic analysis of VBNC *F. tularensis*, which revealed clear differences in gene expression, and we identify sets of differentially regulated genes which are specific to the VBNC state. Identification of these VBNC specific genes will pave the way for future research aimed at dissecting the molecular mechanisms driving entry into the VBNC state.

## Introduction

*Francisella tularensis* is a highly infectious intracellular pathogen that causes the potentially lethal infection, tularemia (Ellis et al., 2002; Keim et al., 2007). It is of particular concern as a potential agent of bioterrorism (Dennis et al., 2001; Maurin, 2015) and has been classified as a Tier 1 Select Agent. *F. tularensis* is widely distributed across the Northern Hemisphere and is divided into two subspecies, *F. tularensis* subsp. *tularensis* and, *F. tularensis* subsp. *holarctica* (Ellis et al., 2002; Sjöstedt, 2007). *F. tularensis* subsp. *tularensis* is endemic to North America, whereas *F. tularensis* subsp. *holarctica* is found throughout the Northern Hemisphere. Both subspecies are known to infect a wide range of hosts, including ∼250 species of animals including lagomorphs, small rodents and various species of reptiles and fish (Mörner, 1992; Ellis et al., 2002; Keim et al., 2007; Sjöstedt, 2007). Biting arthropods are recognized as a key vector of transmission for *F. tularensis* (Petersen et al., 2009), with tick species being a primary vector in North America and biting flies and mosquitoes associated with transmission in Europe (Mörner, 1992; Ellis et al., 2002; Sjöstedt, 2007; Thelaus et al., 2014; Degabriel et al., 2023). Whilst *F. tularensis* subsp. *tularensis* is more virulent, both subspecies of *F. tularensis* can cause infection and severe disease in humans, either through transmission by arthropod vectors or by coming into direct contact with infected animals or contaminated soils or water (Ellis et al., 2002; Sjöstedt, 2007; Degabriel et al., 2023). There is evidence that *F. tularensis* can persist in aquatic environments associated with biofilm (van Hoek, 2013; Schaudinn et al., 2023) or in a free-living state. *F. tularensis* can be detected by molecular methods from aquatic environments, especially from areas that have reported zoonotic outbreaks of tularemia (Kaysser et al., 2008; Berrada and Telford, 2010; Lea Berrada et al., 2011; Sjöstedt et al., 2018). However, the ecology of *F. tularensis* and the factors that influence environmental persistence is not well understood.

Of particular relevance to environmental persistence is the ability of *F. tularensis* to enter into a viable but non-culturable (VBNC) state. The VBNC state is a survival strategy employed by diverse bacteria under conditions of stress, such as nutrient limitation, adverse physical conditions or in the presence of antibiotics (Ramamurthy et al., 2014). Typically, bacteria entering the VBNC state have reduced metabolic activity and are no longer able to be cultured under standard laboratory conditions. Entry into the VBNC state has also been reported to be associated with changes in morphology (Chaiyanan et al., 2007; Krebs et al., 2011; Pawlowski et al., 2011; Casasola-Rodríguez et al., 2013; Shen and Chou, 2016; Kim et al., 2018). Pathogens that are able to enter the VBNC state present a threat for public health and food safety because they are harder to detect by conventional surveillance methods that involve plating or laboratory culture (Ramamurthy et al., 2014).

*Francisella tularensis* has been reported to enter the VBNC state in cold water, in a nutrient limited state, remaining viable for up to 24 weeks (Forsman et al., 2000; Golovliov et al., 2021). Entry into the VBNC state affects the virulence of *F. tularensis*, with *F. tularensis* subsp. *holarctica* retaining the ability to cause disease in a mouse model after 24 weeks in the VBNC state, whereas *F. tularensis* subsp. *tularensis* did not (Forsman et al., 2000; Golovliov et al., 2021). These studies indicate that VBNC *F. tularensis* may be resuscitated by introduction into a mammalian host and subsequently can be cultured on agar plates. However, the molecular mechanisms that drive entry into and resuscitation from the VBNC state for *F. tularensis* are not known and, to date, *in vitro* resuscitation of VBNC *F. tularensis* has not been reported. Understanding the transition into the VBNC state and how the bacteria become resuscitated within hosts will have important implications for our understanding of the epidemiology of *F. tularensis*.

For this study, we used *F. tularensis* LVS, an attenuated strain of *F. tularensis* subsp. *holarctica*, that can be handled safely in BSL2 conditions while retaining pathogenicity in some animal models (Eigelsbach and Downs, 1961; Tigertt, 1962; Elkins et al., 1996). We had observed that, under standard broth culture conditions, *F. tularensis LVS* rapidly and spontaneously enters a VBNC state. Live/dead staining combined with epifluorescence microscopy was used to analyze the progression into the VBNC state and phase contrast and fluorescence microscopy revealed that entry into the VBNC state was accompanied by morphological differentiation. Transmission electron microscopy was used to investigate the ultrastructure of these morphologically differentiated cells. Having established that bacterial cultures older than 1 week had transitioned fully into the VBNC state we wanted to investigate whether these unculturable cells retained any biologically relevant properties. To do this we incubated murine macrophages with VBNC bacteria and measured production of TNF-α and used fluorescently labeled bacteria to investigate their uptake and persistence in these host cells. We also found that VBNC *F. tularensis* LVS was capable of invading human erythrocytes at levels comparable to culturable bacteria and used a gentamicin susceptibility assay to show that VBNC *F. tularensis* LVS was insensitive to this antibiotic. Finally, the results of a transcriptomic analysis of VBNC *F. tularensis* LVS presented here demonstrate active transcription in these dormant bacteria and altered patterns of gene expression between the culturable and VBNC states.

## Materials and methods

### Bacterial strains, plasmids, and growth conditions

Bacterial strains used in this study are listed in Table 1. For cultivation of *F. tularensis* LVS strains, frozen stock cultures of bacteria were streaked onto chocolate II agar plates (Horzempa et al., 2011) and incubated at 37°C with 5% CO_2_ for 3–7 days. For broth cultures, bacteria from chocolate II agar plates were used to inoculate either Chamberlain’s chemically defined medium (CDM; pH 6.3) (Chamberlain, 1965), tryptic soy broth (Becton, Dickinson, and Co.) supplemented with 0.1% cysteine hydrochloride (TSBc; Fisher Scientific and grown to stationary phase by overnight incubation at 37°C with agitation). Where required, media were supplemented with antibiotics at the following concentrations: kanamycin (10 μg ml^–1^). Drip plating was used to determine the CFU ml^–1^ for culturable *F. tularensis* LVS and VBNC bacteria, for which drip plating was not possible, were adjusted to the same OD_600_ as the culturable bacteria in all experiments.

**TABLE 1:** Bacterial strains, plasmids, and oligonucleotides used in this study.

	Description	Source or references
***F. tularensis* strains**		
LVS	*Francisella tularensis* subsp. *holarctica* live vaccine strain	Karen Elkins
LVS pSC13	LVS containing promoterless *emgfp* on pSC13	Cantlay et al., 2020
LVS pKHEG	LVS expressing *emgfp*	Cantlay et al., 2020
LVS pTC3D	LVS expressing *tdtomato*	Horzempa et al., 2008
LVS *iglC*-null	LVS with both copies of *iglC* deleted	Schmitt et al., 2017
LVS *iglC*-null pKHEG	LVS with both copies of *iglC* deleted and expressing *emgfp*	D’Cunha, 2023
**Plasmids**		
pSC13	*emgfp* cloned into the *Nde*I *Bam*HI site of pFNLTP8	Cantlay et al., 2020
pKHEG	326 nucleotides, including *FGRp*, from pTC3D cloned into the *Kpn*I *Nde*I site of pSC13	Cantlay et al., 2020
pTC3D	pF8tdTomato with the FGRp from the LVS chromosome cloned upstream of tdtomato	Horzempa et al., 2008

### Phase contrast and epi-fluorescence microscopy of morphological differentiation

Wild type *F. tularensis* LVS was cultured in CDM for 24, 48, 96, and 336-h in triplicate. At each time point 1 ml of bacteria were incubated for 30 min with 10 μg ml^–1^ FM 4–64 fluorescent dye before being spotted onto pads of 1% agarose in PBS. Fluorescently labeled bacteria were spotted onto pads of 1% agarose in PBS and imaged using an Olympus IX73 microscope equipped with a 100x NA. 1.30 Phase objective and an ORCA-Flash4.0 LT+ Digital CC11440-42U CMOS camera (Hamamatsu). Exposure times of 50 ms were used for each channel, and images were processed using ImageJ (Schneider et al., 2012) where the dark and light points were set to the same levels for all images. Bacteria that had not been stained with FM 4–64 were used as a negative control for fluorescence.

### Circularity measurements for *F. tularensis* LVS cells

Cultures of *F. tularensis* LVS were grown in TSBc medium at 37°C for 24, 48, 72, 216, 336, 504, and 672 h. At each time point 10 μl of culture medium was spotted onto pads of 1% agarose in PBS and visualized using an Olympus IX73 microscope equipped with a 100x NA. 1.30 Phase objective and an Olympus XM10 CCD camera. The perimeter and skeletal length of bacteria were measured in ImageJ (Schneider et al., 2012) and a circularity score was assigned with a score of one indicating perfect circularity. Each field of view was analyzed using the following steps in ImageJ: Analyze >> Set Measurements >> Check the following: Area, Shape Descriptors, and Perimeter. Set “Decimal places” to 3, Protocol: 1. Process >> Find Edges 2. Process >> Smooth 3. Process >> Binary >> Make Binary 4. Process >> Binary >> Fill Holes 5. Analyze >> Analyze Particles >> set Size (pixel^2): to 50–150 Circularity to 0.7–1.00. For each timepoint, 3 fields of view were analyzed from 2 independent cultures with at least 2200 bacteria being measured. Exposure times of 50 ms were used for each channel, and images were processed using ImageJ (Schneider et al., 2012), dark and light points were set to the same levels for all images.

### Live/dead staining of *F. tularensis* LVS

*Francisella tularensis* LVS was grown for 24, 48, 168, 336, 504, and 672 h in TSBc medium at 37°C. At each time point, 100 μl of bacterial cell suspension was mixed with an equal volume of the 2X stock solution from the LIVE/DEAD^®^ BacLight™ Bacterial Viability Kit (Invitrogen) to a final concentration of 6 μM SYTO 9 and 30 μM propidium iodide and incubated for 15 min in the dark. A total of 10 μl of culture medium was spotted onto pads of 1% agarose in PBS and fluorescence was visualized using an Olympus IX73 microscope equipped with a 100x NA. A total of 1.30 Phase objective and an Olympus XM10 CCD camera. Exposure times of 50 ms were used for each channel, and images were processed using ImageJ (Schneider et al., 2012) dark and light points were set to the same levels for all images. Bacteria that had been heat killed at 80°C for 20 min were used as a control for dead bacteria. Bacteria that had not been stained were used as a negative control for fluorescence. The proportion of live and dead bacteria was manually scored, 3 fields of view were analyzed from 2 independent cultures for each time point with at least 1000 bacteria being measured in each case.

### Culturability assays of *F. tularensis* LVS transitioning into the VBNC state

Drip plating was used to measure culturability. Wild type *F. tularensis* LVS grown on chocolate agar plates were resuspended in either TSBc or CDM, the OD_600_ was measured (2.15 and 2.5, respectively) and was diluted 100-fold to inoculate 10 ml cultures of TSBc or CDM. At intervals of 24 h, a small aliquot was removed from each culture, serially diluted (10^–2^ to 10^–8^) in PBS and drip plated onto chocolate agar to enumerate CFUs every 24 h for 7 days. After 1 week, CFUs were enumerated again at the 2-week and then 3-week time points. Drip plating was done in triplicate and, at least up until the 120 h time point, sufficient colonies on the plates were obtained to accurately estimate the CFU ml^–1^ (Jennison and Wadsworth, 1940; Glasson et al., 2016). After 120 h the number of colonies on the plates fell below the limit of quantification and by 168 h (7 days) we did not observe any CFU. We also plated 100 μl of culture medium at the later time points, up to 8 weeks of incubation, and did not observe any CFU.

### Transmission electron microscopy

Wild type *F. tularensis* LVS was cultured in CDM for 24, 48, 96, and 336-h in triplicate. At each time point, 1 ml of cells was pelleted from the same flask and was fixed for 1 h in 2.5 % glutaraldehyde in 0.1 M Sorensen’s buffer, pH 7.2 (Electron Microscopy Sciences) and then washed twice in 0.1 M Sorensen’s buffer, pH 7.2 (Electron Microscopy Sciences) and stored at 4°C. Washed bacteria were fixed in 1% OsO_4_, 1% K_3_Fe (CN)_6_ for 1 h. Following 3 additional PBS washes, the pellet was dehydrated through a graded series of 30–100% ethanol, 100% propylene oxide then infiltrated in 1:1 mixture of propylene oxide: Polybed 812 epoxy resin (Polysciences, Warrington, PA, USA) for 1 h. After several changes of 100% resin over 24 h, pellet was embedded in a final change of resin, cured at 37°C overnight, followed by additional hardening at 65°C for two more days. Ultrathin (60–70 nm) sections were collected on 200 mesh copper grids, stained with 2% uranyl acetate in 50% methanol for 10 min, followed by 1% lead citrate for 7 min. Sections were imaged using a JEOL FLASH JEM 1400 transmission electron microscope (Peabody, MA) at 80 kV fitted with a bottom mount AMT 2k digital camera (Advanced Microscopy Techniques, Danvers, MA, USA).

### ELISA analysis of TNF-α production in RAW 264.7 murine macrophages challenged with culturable and VBNC *F. tularensis* LVS, *in vitro*

To assay production of TNF-α, 5 × 10^4^ RAW 264.7 murine macrophage cells were seeded in Primaria-coated 96-well plates and incubated for 2 h at 37°C with 5% CO_2_ with wild type *F. tularensis* LVS that had been grown in TSBc medium for either 24 h (culturable) or 336 h (VBNC) at a MOI of 10. VBNC bacteria cultured for 336 h in TSBc were heat killed at 80°C to test if the effect on TNF-α observed for VBNC bacteria required that they be alive. Additionally, RAW 264.7 cells not incubated with bacteria was used as a negative control. Supernatants were analyzed using a Mouse TNF-α DuoSet ELISA kit (RandD Systems) according to the manufacturer’s instructions. Standards were assayed in triplicate and experimental samples from three biological replicates were analyzed.

### Macrophage infection assays

1 x10^5^ RAW 264.7 cells cultured in Dulbecco’s Modified Eagle’s Medium (DMEM) (Gibco) supplemented with 10% FBS (Gemini Bioproducts), 25 mM HEPES (Corning), 2 mM Glutagro (Corning) and 1 mM Sodium Pyruvate (Gibco) were seeded in glass bottomed 35 mm dishes (Ibidi, GmbH) and incubated at 37°C with 5% CO_2_ to give confluent growth after 24 h. Wild type *F. tularensis* LVS containing *emgfp* on the plasmid pKHEG (Cantlay et al., 2020) was cultured in CDM for 24 h (Culturable) and 720 h (VBNC). An LVS *iglC-*null strain, lacking the T6SS and blocked in intracellular replication (Schmitt et al., 2017), transformed with pKHEG was cultured for 24 h in CDM and used as a negative control for intracellular replication. Bacteria were diluted in supplemented DMEM and incubated with the RAW 264.7 macrophages at an MOI of 100 for 24 h. Macrophages were washed twice in PBS and fresh supplemented DMEM was added, and the cells were imaged directly in the 35 mm dishes. Phase contrast and epifluorescence images were captured with an Olympus IX73 microscope equipped with a 100x NA. 1.30 Phase objective and an ORCA-Flash 4.0 LT+ Digital CC11440-42U CMOS camera (Hamamatsu). Exposure times of 50 ms were used for each channel, and images were processed using ImageJ (Schneider et al., 2012). All fluorescence images were adjusted for contrast and brightness using RAW 264.7 cells infected with wild type, culturable *F. tularensis* LVS containing the empty vector pSC13 and RAW 264.7 cells not incubated with bacteria as controls for natural auto fluorescence.

### Double immunofluorescence microscopy to determine invasion and attachment in human erythrocytes

*Francisella tularensis* LVS bacteria were incubated in CDM at 37°C for either 24 h (Culturable) or 840 h (VBNC), resuspended in pre-warmed RBC medium and mixed with 1 × 10^6^ erythrocytes to an MOI of 100. Erythrocytes and bacteria were incubated together for 2 h. Cells were pelleted at 100 × g for 5 min and washed twice in PBS. Cells were then fixed in 2% paraformaldehyde and 0.1% glutaraldehyde in PBS for 1 h at room temperature. Fixed cells were washed 3 times in PBS and then blocked in 2.5 % BSA in PBS for 30 min at room temperature. Cells were probed with polyclonal mouse anti-*F. tularensis* Mab1 antibody (BEI Resources) at a concentration of 1:1000, overnight at 4°C. Cells were washed 3 times in PBS 0.05% Tween 20 and probed with Alexa Fluor 360 Donkey anti-Mouse IgG (Invitrogen) at a concentration of 1:1000 for 1 h at room temperature. Cells were washed 3 times in PBS 0.05% Tween 20 and erythrocyte membranes were permeabilized with 0.1% Triton-X for 10 s, spun down for 5 min at 100 × g and then washed 3 times in PBS. Cells were re-blocked in 2.5 % BSA in PBS for 30 min at room temperature and then were re-probed with polyclonal mouse anti-*F. tularensis* Mab1 antibody (BEI Resources) at a concentration of 1:1000 for 1 h at room temperature. Cells were washed 3 times in PBS 0.05% Tween 20 and probed with Alexa Fluor 488 Rat anti-Mouse IgG (Invitrogen) at a concentration of 1:1000 for 1 h at room temperature. Cells were washed 3 times in PBS 0.05% Tween 20 and then washed once in PBS. Cells were spotted on pads of 1% agarose in PBS for imaging. Experiments were performed in triplicate and at least 10 fields of view were analyzed for each condition. A total of 914, 552 and 784 RBCs were scored for culturable wild type, culturable *iglC*-null and VBNC wild type bacteria, respectively. Bacteria that were dual labeled were scored as having attached, and bacteria with only the green fluorescence were scored as having invaded.

### Gentamicin sensitivity assay

*Francisella tularensis* LVS cells expressing *emgfp* were cultured in CDM for either 24 h (Culturable) or for 504 h (VBNC) at 37°C. Bacteria were normalized by optical density and diluted in fresh growth medium. Six replicates were aliquoted in a 96-well plate for incubation with shaking at 37°C with or without 100 μg/ml gentamicin in a BioTek Synergy H1 plate reader. Optical density and fluorescence were measured at 2-h intervals. To obtain fluorescence micrographs, *F. tularensis* LVS bacteria expressing *emgfp* were cultured in CDM for either 24 h (Culturable) or for 552 h (VBNC) at 37°C. Cultures were then incubated for 1 h with (+) or without (-) 100 μg ml^–1^ gentamicin. Bacteria were spotted onto pads of 1% agarose in PBS and imaged using an Olympus IX73 microscope equipped with a 100x NA. 1.30 Phase objective and an ORCA-Flash4.0 LT+ Digital CC11440-42U CMOS camera (Hamamatsu). Exposure times of 50 ms were used for each channel, and images were processed using ImageJ (Schneider et al., 2012) where the dark and light points were set to the same levels for all images. *F. tularensis* LVS containing the empty vector pSC13 was used as a control for fluorescence.

### Oxford nanopore transcriptomics

Wild type *F. tularensis* LVS was cultured in CDM at 37°C for 24, 48, 96, and 336 h in triplicate (T24, T48, T96, T336). At each time point, cultures were standardized to an OD_600_ of 1 and 10 ml of culture was pelleted, snap frozen and stored at −80°C before RNA extraction. RNA was extracted using Trizol (Invitrogen) and Phasemaker™ tubes (Invitrogen) according to the manufacturer’s instructions. The supernatants were loaded onto PureLink™ RNA Mini (Invitrogen) spin columns, and processed according to the manufacturer’s instructions, including an on-column DNase step using PureLink™ DNase Mixture (Invitrogen). Purified RNA was analyzed using a NanoDrop ND1000 spectrophotometer (Thermo Fisher). A total of 1 μg RNA was processed using the MICROBExpress™ Bacterial mRNA Enrichment Kit (Invitrogen). For each timepoint, 50 ng of ribosomally depleted mRNA was used to generate a barcoded cDNA library with the PCR-cDNA Barcoding Kit (SQK-PCB109—Oxford Nanopore Technologies) according to the manufacturer’s instructions. The barcoded cDNA library was loaded onto a FLO-MIN106 flow cell and sequencing was performed for 44 h on a Linux Ubuntu desktop with the MinKNOW software version 20.06.5.

Basecalled and demultiplexed fastq files passing minimum quality filters (MinKNOW defaults) were aggregated into single files for each sample and mapped to the *F. tularensis* subsp. *holarctica* LVS reference genome (ASM924v1) using the long read preset option (-x map-ont) in Minimap2 (Li, 2018). Mapped reads were filtered, sorted and indexed using SAMtools (Danecek et al., 2021). Aligned reads were quantified using HTSeq2 (Givanna et al., 2022), generating raw counts for each time point and biological replicate. The DESeq2 (Love et al., 2011) pipeline was used to identify differentially expressed genes DEGs, with a focus on the T24/T336 contrast.

### Illumina transcriptomics

Wild type *F. tularensis* LVS was cultured in CDM at 37°C for 24, 48, 96, 240, and 336 h. At each time point, cultures were standardized to an OD_600_ of 1 and 10 ml of culture was pelleted, snap frozen and stored at −80°C before RNA extraction. RNA was extracted using TRIzol reagent (Invitrogen) and Phasemaker™ tubes (Invitrogen) according to the manufacturer’s instructions. The supernatants were loaded onto PureLink™ RNA Mini (Invitrogen) spin columns, and processed according to the manufacturer’s instructions, including an on-column DNase step using PureLink™ DNase Mixture (Invitrogen). Purified RNA was analyzed using an Agilent 2100 Bioanalyzer. Prior to library construction, rRNA was removed from RNA samples using the Thermo Fisher MICROBExpress kit; RNA libraries were prepared from ribo-depleted RNA using the Illumina TruSeq Stranded RNA Library Prep kit. Libraries were sequenced in a 2 × 50 base paired end strategy on an Illumina NextSeq2000 sequencer on a P2 flow cell. High quality reads were mapped to the *F. tularensis* subsp. *holarctica* LVS reference genome (ASM924v1) with HISAT2 (Kim et al., 2019), reads were quantified using HTSeq2 (Givanna et al., 2022), and differential expression analysis was done with DEseq2 (Love et al., 2011). The T24/T336 comparison was of primary interest and allowed for comparison of transcriptional changes between culturable and VBNC forms of *F. tularensis* across both sequencing methodologies.

### Statistical analyses

Data were analyzed using GraphPad Prism software. The tests used and the *P*-values obtained are presented in the text and figure legends. Asterisks are used in the figures to indicate statistical significance; * denotes *p* < 0.05, ** denotes *p* < 0.01 and *** denotes *p* < 0.001.

## Results

### *F. tularensis* LVS undergoes morphological differentiation under standard broth culture conditions

**FIGURE 1:**
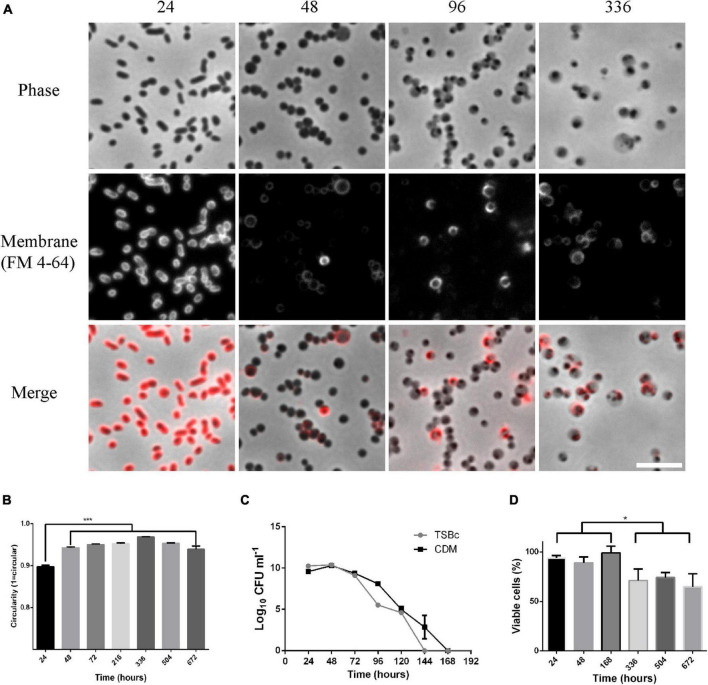
Morphological changes observed for *Francisella tularensis* LVS cultures coincide with a transition to a viable but non-culturable (VBNC) state. **(A)** Cultures of *F. tularensis* LVS were grown in CDM at 37°C for the times indicated. Cultures were further incubated with 10 μg ml^–1^ FM 4–64 for 30 min before being spotted onto pads of 1% agarose in PBS and visualized by phase contrast (**top** panel) and epifluorescence (**center** panel) microscopy. The merge of the phase contrast and fluorescence images is shown on the **lower** panel. At the 24-h time point, cells have pleomorphic, coccobacillus morphology and stain evenly for the membrane dye FM 4-64. After 48 h, bacterial cells have a round appearance and membrane staining is uneven. After 96 h cells appear phase light with one or more phase dark regions and stain unevenly for FM 4–64. Scale bar represents 5 μm. **(B)** Cultures of *F. tularensis* LVS were grown in TSBc medium at 37°C for the times indicated and spotted onto pads of 1% agarose in PBS and visualized by phase contrast microscopy. The perimeter and skeletal length of bacteria were measured in ImageJ and a circularity score was assigned with a score of one indicating perfect circularity. Populations of cells incubated for 48 h or longer were significantly rounder than bacteria cultured for 24 h (*p* < 0.001 determined by ordinary 1-way ANOVA with Tukey’s multiple comparisons test). For each timepoint, 3 fields of view were analyzed from 2 independent cultures with at least 2200 bacteria being measured. Bars show the standard error. **(C)** *F. tularensis* LVS was grown in TSBc or CDM at 37°C and drip plating assay on chocolate agar was used to enumerate culturable cells. No colonies are detected from cultures incubated for 7 days or longer. The average of 3 independent cultures is shown. Error bars show the standard deviation. **(D)** The viability of *F. tularensis* cultures grown in TSBc at 37°C for the time points indicated was determined using the BacLight live dead staining kit. A drop in viability is observed in cultures grown for 336 h or longer (*p* < 0.05 determined by unpaired *t*-test), however, even up to 672 h (4 weeks) incubation ∼70% of bacteria remained viable. The percentage of viable cells at each time point was calculated from 3 fields of view of 2 independent cultures and at least 1000 cells were counted for each time point. Error bars show the standard error. The asterisks denote significance, * denotes *p* < 0.05 and *** denotes *p* < 0.001.

Sub-culture of *F. tularensis* LVS grown in broth in the laboratory becomes less efficient after 3 days of growth and after between 4 and 7 days becomes impossible. To learn more about the physiological changes that occur as *F. tularensis* LVS entered this unculturable state we made microscopic observations of the bacteria at different time points during the process. Cultures were grown in CDM, and the lipid membrane was labeled with FM 4-64 dye and analyzed by epifluorescence microscopy. Bacteria grown to stationary phase by incubation for 24 h appeared as small pleomorphic coccobacilli with even membrane staining. This is the expected morphology of *F. tularensis* LVS and the morphology that has most commonly been reported. After 48 h of growth, a pronounced change in cellular morphology was observed with cells appearing larger and rounder. For bacteria grown for 48 h or longer membrane staining became uneven, with some cells staining very brightly and others hardly at all. Bacteria at 48 h appeared phase dark but were of varying sizes. After 96 h incubation bacteria appeared as phase light but with one or more phase dark spots. On prolonged incubation, such as for 336 h, most cells appeared large and phase light with small dark spots and some bacteria appeared small, round and phase dark (Figure 1A). Cultures that had been incubated for longer times (up to 6 weeks) were routinely imaged and the same morphologies were observed. Membrane and nucleic acid staining of bacteria incubated in broth culture (TSBc) for 5 weeks showed that membrane and DNA were maintained in these cells and bacteria that produced TdTomato, heterologously on the plasmid pT3CD (Horzempa et al., 2008), maintained fluorescence (Supplementary Figure 1). Interestingly, *F. tularensis* LVS cultures that have been allowed to dry out and left at room temperature under ambient conditions for extended periods of time (greater than 1 year) can be resuspended in PBS or growth medium and retain similar morphologies, suggesting that these large, phase light cells with phase dark spots can be very durable (Supplementary Figure 2). To further quantify the change in shape that occurred, phase contrast micrographs of bacteria grown in TSB were analyzed in ImageJ by measuring the perimeter and skeletal length of cells which generated a circularity score, with 1 representing a perfect circle. Cultures incubated for between 48 and 672 h (4 weeks) were analyzed and were all found to be significantly rounder than bacteria from 24-h old cultures (*p* < 0.001 determined by ordinary 1-way ANOVA with Tukey’s multiple comparisons test) but no significant difference in cell roundness was found between these cultures (Figure 1B). These results suggest that *F. tularensis* cells undergo a significant change in shape after 48 h of broth culture, and that this change in shape is a terminal phenotype.

### *F. tularensis* LVS spontaneously enters a viable but non-culturable state in broth culture

The spontaneous transition from small irregular rods to rounder, often larger cells, coincided with the transition into the non-culturable state. To try to understand if the two phenomena were linked in any way, we conducted an investigation of both culturability and viability in broth cultures of *F. tularensis* LVS. To investigate culturability, serial dilution and drip plating was used to measure the plating efficiency of *F. tularensis* LVS in broth culture. Plating efficiency is high in cultures grown in either CDM or TSBc for 24–48 h. By 96 h plating efficiency starts to fall, and after this time point CFU fell precipitously. The 96 h time-point appears to represent an inflection point at which the onset of the VBNC state begins and also coincides with the change in microscopic appearance of the bacterial cells from phase dark to phase light. After 7 days incubation, no CFU were recovered at any of the time points tested (Figure 1C). Drip plating experiments in which 10 μl of culture medium was used have a theoretical detection limit of 100 CFU/ml. However, we also routinely plated out 100 μl of VBNC cultures or used this same volume as an inoculum for broth cultures, lowering the theoretical limit of detection to 10 CFU/ml. Whilst we cannot absolutely rule out the possibility that there were viable cells below this theoretical limit of detection, dozens of attempts to resuscitate cultures after 7 days incubation were all unsuccessful. These results indicate that, regardless of indicators of viability (such as DNA, intact membranes and cytosolic fluorescent proteins), the bacteria were unculturable after this 7-day period. To investigate viability, the percentage of cells with intact membranes was determined using Live/Dead staining with epifluorescence microscopy to measure the viability of the bacteria over the same time frames. The viability of bacteria for up to 1 week in broth culture was approximately 90% and, although viability dropped slightly after this (*p* < 0.05 determined by unpaired *t*-test), approximately 70% of cells remained viable at the subsequent time points of 2, 3, and 4 weeks (Figure 1D).

*Francisella tularensis* LVS reproducibly and spontaneously entered a VBNC state after 3–4 days growth in broth culture and that this VBNC state is maintained for at least 4 weeks. Testing culturability and viability for longer periods was not routinely performed because cultures would dry out over extended periods. In fact, it was attempts to add medium and re-culture the dried-out bacteria that led to observations that the morphology of the cells remained consistent, even after extended periods of desiccation.

These results show that *F. tularensis* LVS cells rapidly and spontaneously undergo morphological and physiological differentiation under standard broth culture conditions. Interestingly, the initial change in shape observed after 48 h does not coincide with the transition into the VBNC state as these bacteria plate at high efficiency. However, the appearance of phase light and phase dark regions of the bacterial cell observed after 96 h appears to be coincident with the transition into the VBNC state.

### Outer membrane detachment is observed in morphologically differentiated cells and increases in frequency in older cultures

**FIGURE 2:**
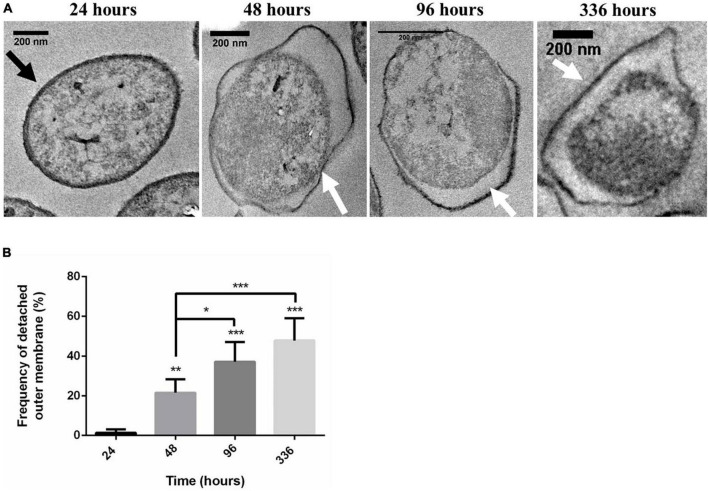
Viable but non-culturable state (VBNC) *Francisella tularensis* LVS cells have an increased frequency of detached outer membranes. **(A)** Cells incubated in CDM at 37°C were fixed in 2.5% glutaraldehyde at the indicated time points and imaged by TEM. A black arrow indicates a typical membrane of a viable cell and white arrows indicate the detached membranes observed in morphologically differentiated cells after 48, 96, or 336-h. Scale bars equal 200 nm. **(B)** The frequency of detached outer membranes was measured at the indicated time points. For each condition, 5 fields of view were analyzed and at least 592 cells were measured for each time point. There were significantly more detached outer membranes after 336-h (VBNC) compared to the 24-h (culturable) time point (*p* < 0.001 determined by ordinary 1 way ANOVA with Dunnett’s multiple comparisons test). There was also a significant difference between the 24 and 48-h timepoints (*p* < 0.01 determined by ordinary 1 way ANOVA with Dunnett’s multiple comparisons test) and between the 48-h time point and the 96 and 336-h time points (*p* < 0.001 determined by ordinary 1 way ANOVA with Dunnett’s multiple comparisons test). The asterisks denote significance, * denotes *p* < 0.05, ** denotes *p* < 0.01, and *** denotes *p* < 0.001.

To better understand the morphological changes observed, transmission electron micrographs were obtained for cells grown in CDM at four time points, 24-h (corresponding to culturable and non-differentiated), 48-h (a time point where cells had morphologically differentiated but were still culturable), 96-h (a time point at which most cells had transitioned into a VBNC state), and 336-h (a time point where all cells were either non-viable or in the VBNC state) (Figure 2A). Culturable cells at the 24-h time point had a low frequency of detached outer membranes (1.3%). Whereas culturable but morphologically differentiated cells at the 48-h time point had significantly more (21.7%; *p* < 0.01 determined by ordinary 1 way ANOVA with Dunnett’s multiple comparisons test). The frequency of detached outer membranes increased with incubation time with 37.2% observed at the 96-h time point and 47.9% observed at 336 h (*p* < 0.001 determined by ordinary 1 way ANOVA with Dunnett’s multiple comparisons test) (Figure 2B). Detached outer membranes were the most notable feature revealed by the electron micrographs, however, particularly at the 336-h time point the cells had distinct electron dense areas which could be consistent with the phase dark spots observed in the phase contrast micrographs. The phase light areas observed in the light micrographs, then, may correspond to the more electron lucent areas of the cells (Supplementary Figure 3). It is likely that detachment of the outer membrane is a significant factor contributing to the observed morphological changes. Given that neither culturability nor viability is significantly diminished at the 48-h time point it is reasonable to assume that outer membrane detachment does not significantly affect viability of these cells.

### VBNC *F. tularensis* LVS bacteria interact with host macrophage cells *in vitro*

We wanted to investigate if VBNC *F. tularensis* LVS retained any biological activity, such as an ability to interact with potential host cells. For this we conducted gentamicin protection, infection assays with RAW 264.7 mouse macrophages as described previously (Cantlay et al., 2022). Macrophages were incubated with *F. tularensis* LVS bacteria grown for either 24 h (culturable) or 336 h (VBNC) for 2 h, after which the macrophages were washed and incubated with gentamicin to kill any extracellular bacteria. Macrophages were lysed at time intervals after infection and intracellular bacteria were enumerated by drip plating. However, VBNC bacteria did not replicate in the macrophages and no CFU were obtained indicating that incubation with host cells is not sufficient for resuscitation of VBNC *F. tularensis* LVS under the conditions tested.

**FIGURE 3:**
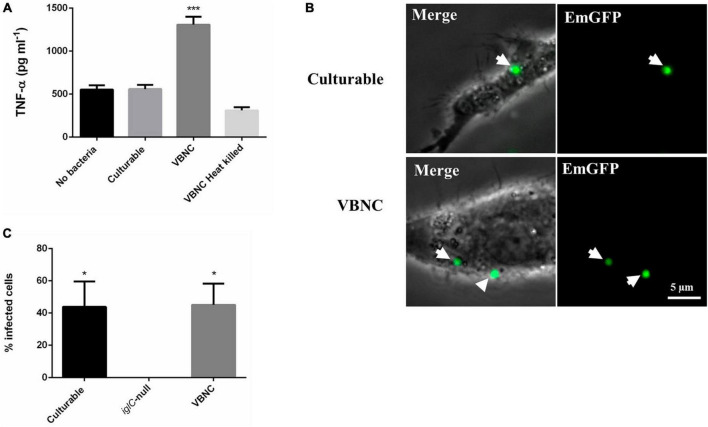
Viable but non-culturable state (VBNC) *Francisella tularensis* LVS stimulates production of TNF-α in RAW 264.7 mouse macrophages. Whilst they do not proliferate in RAW 264.7 cells they are not efficiently cleared. **(A)** 5x10^4^ RAW 264.7 cells were incubated for 2 h with wildtype *F. tularensis* grown in TSBc medium at 37°C for 24 (Culturable) and 336 (VBNC) hours at an MOI of 10. RAW 264.7 incubated with heat killed, 336-h old VBNC bacteria or no bacteria were used as controls. TNF-α production was significantly higher in RAW 264.7 macrophages incubated with VBNC *F. tularensis* compared to the other conditions (*p* < 0.001 determined by ordinary 1 way ANOVA with Dunnett’s multiple comparisons test). Error bars show the standard deviation from the mean of three biological replicates. **(B)** 1 x10^5^ RAW 264.7 cells were seeded in 35 mm dishes to give confluent growth after 24 h. Cells were then incubated with EmGFP labeled *F. tularensis* LVS strains at an MOI of 100 for 24 h. Both culturable and VBNC bacteria were observed inside macrophages (white arrows). Scale bar equals 5 μm. **(C)** RAW 264.7 cells were then scored for the presence of intracellular bacteria. The mean number of bacteria per macrophage was similar for culturable and VBNC wild type bacteria and significantly different from an *iglC*-null strain (grown for 24 h in CDM) for which no fluorescently labeled bacteria were observed, determined by 1-Way ANOVA *p* < 0.05. Error bars show the standard error of the mean from 10 fields of view and at least 35 macrophages were scored for each condition. The asterisks denote significance, * denotes *p* < 0.05 and *** denotes *p* < 0.001.

To test if macrophage cells were able to respond when incubated with VBNC bacteria we measured the production of the pro-inflammatory cytokine TNF-α. RAW 264.7 macrophages were incubated with *F. tularensis* LVS bacteria grown for either 24 h (culturable) or 336 h (VBNC) at an MOI of 10 for 2 h and TNF-α production was measured by ELISA. Macrophages incubated with culturable bacteria produced a similar amount of TNF-α as macrophages that were not exposed to bacteria (Figure 3A). TNF-α was significantly upregulated (∼2.5 fold) in macrophages incubated with VBNC bacteria (*p* < 0.001 determined by ordinary 1 way ANOVA with Dunnett’s multiple comparisons test). This suggests that the RAW 264.7 macrophages were capable of sensing and reacting to the VBNC bacteria whereas culturable bacteria were able to evade the host cells’ proinflammatory response. To test if the upregulation of TNF-α required an active process, such as secretion of a protein or effector molecule, we used heat killed VBNC bacteria as a control. Interestingly, when macrophages were exposed to VBNC bacteria that had been heat killed, TNF-α production was restored to levels seen for either culturable bacteria or for cells not exposed to bacteria (Figure 3A).

Next, we used epi-fluorescence microscopy to investigate whether the RAW 264.7 macrophages were able to phagocytose VBNC bacteria. For this, *F. tularensis* LVS strains expressing Emerald Green Fluorescent Protein (EmGFP) on the plasmid pKHEG (Cantlay et al., 2020) were incubated with RAW 264.7 murine macrophages for 24 h. Macrophages were then washed to remove any extracellular bacteria before imaging. For RAW 264.7 cells incubated with culturable bacteria, the majority of the macrophage cells had been killed due to infection by an intracellular replication of the bacteria. In contrast, there was no sign that VBNC bacteria were able to kill the macrophage cells. Additionally, we tested an LVS *iglC*-null strain, that is defective in intracellular replication (Schmitt et al., 2017) but is not affected in culturability or viability. After co-culture for 24 h, as expected, no killing of the macrophages was observed (Supplementary Figure 4). This result was consistent with the gentamicin protection assays which showed that VBNC bacteria do not proliferate within RAW 264.7 macrophages. However, fluorescently labeled VBNC *F. tularensis* LVS was observed co-localizing with macrophage cells, indicating that they are taken up by the macrophages (Figure 3B). We measured the proportion of macrophage cells that contained fluorescent bacteria after 24 h. In the case of culturable bacteria, a large proportion of the cells were killed, however, 43.9% of the remaining cells contained at least one fluorescent bacterium. For VBNC bacteria, the proportion of macrophages with at least one fluorescent bacterium was 45.1%, suggesting that VBNC bacteria are efficiently taken up by the RAW 264.7 macrophages (Figure 3C). No fluorescently labeled *iglC*-null bacteria were observed in the RAW 264.7 macrophages (Supplementary Figure 4). This is not surprising as this strain lacks a functioning Type Six Secretion System which is required for efficient escape from the phagolysosome and so presumably these bacteria are efficiently cleared from the macrophages. That VBNC bacteria can be seen inside the RAW 264.7 cells after 24 h suggests that they are able to either evade or resist clearance by the macrophages.

### VBNC bacteria can attach to and invade human erythrocytes with the same efficiency as culturable bacteria

**FIGURE 4:**
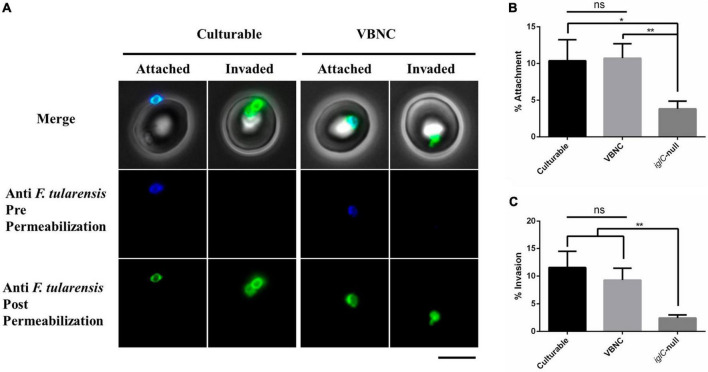
Viable but non-culturable state (VBNC) *Francisella tularensis* LVS attaches to and invades human erythrocytes at the same rate as culturable *F. tularensis LVS*. A total of 1 × 10^6^ Red Blood Cells (RBCs) were incubated for 24 h with culturable (24 h old) and VBNC (840 h old) wild type *F. tularensis* strains at an MOI of 100. **(A)** After 2 h, RBCs were washed in PBS and fixed in 2% paraformaldehyde and 0.1 % glutaraldehyde. Fixed RBCs were probed with an anti-*F. tularensis* primary antibody and a secondary antibody conjugated to blue fluorescent Alexa 350. RBCs were then permeabilized with Triton-X and re-probed with the anti-*F. tularensis* primary antibody and a secondary antibody conjugated to green fluorescent Alexa 480. Bacteria that are attached to RBCs are labeled with both blue and green fluorescence whereas any bacteria that successfully invaded an RBC will only be labeled, after permeabilization, with green fluorescence. For both culturable and VBNC bacteria, examples of attached (blue and green) and invaded (green only) bacteria are shown, Scale bar equals 5 μm. RBCs were scored for attached **(B)** and invaded **(C)** bacteria. Both culturable and VBNC bacteria were observed to be attached to RBCs at a higher rate than the *iglC*-null strain, *p* < 0.05 and 0.001, respectively, as determined by unpaired *t*-test with Welch’s correction. Significantly more invasion was observed for culturable and VBNC bacteria as compared to the *iglC*-null strain, *p* < 0.001 as determined by unpaired *t*-test with Welch’s correction. There was no significant difference in either attachment or invasion of culturable compared to VBNC bacteria. The means of 3 independent experiments are shown. A total of 914, 552 and 784 RBCs were scored for culturable, *iglC*-null and VBNC bacteria, respectively. Error bars show the standard deviation. The asterisks denote significance, * denotes *p* < 0.05 and ** denotes *p* < 0.01.

*Francisella tularensis* LVS can invade red blood cells and may do so in order to evade host immune responses or it may be a mechanism to persist in vectors such as ticks or biting arthropods (Horzempa et al., 2011; Schmitt et al., 2017). Entry into the VBNC state may be promoted by the same conditions that are likely to be encountered in the gut of an arthropod or under the stressful conditions in a host. To test if VBNC cells are able to interact with red blood cells, double immunofluorescence microscopy (DIFM) was used. For this, bacteria grown for either 24 h (culturable) or for 840 h (VBNC) in CDM were incubated with red blood cells for 24 h at an MOI of 100. Erythrocytes were washed to remove extracellular bacteria and then fixed. Erythrocytes were probed with an antibody specific to *F. tularensis* and a secondary antibody conjugated to a blue fluorescent dye. Erythrocytes were then permeabilized and re-probed with the same anti-*F. tularensis* antibody and a secondary antibody conjugated to a green fluorescent dye. In this way, it is possible to distinguish between bacteria that are attached to the red blood (which will fluoresce both blue and green) and those that have invaded the red blood cell (which fluoresce only green) (Figure 4A). For erythrocytes incubated with culturable or VBNC bacteria there was no significant difference in the frequency of attachment with 10.4 and 10.7% respectively. The *iglC*-null strain was used as a control in these experiments as this strain is also defective in erythrocyte invasion (Schmitt et al., 2017). For erythrocytes incubated with culturable *iglC*-null *F. tularensis* LVS there was significantly less attachment, 3.8% (*p* < 0.05 and 0.001 for culturable and VBNC bacteria, respectively, as determined by unpaired *t*-test with Welch’s correction) (Figure 4B). A similar result was seen for bacterial invasion of erythrocytes with culturable bacteria seen invading at a rate of 11.5% and VBNC bacteria invading at a rate of 9.3%. The rate at which both culturable and VBNC wild type bacteria invaded erythrocytes was significantly greater than the rate seen for the *iglC*-null strain (*p* < 0.001 as determined by unpaired *t*-test with Welch’s correction) (Figure 4C). These results indicate that despite being unculturable under standard laboratory conditions, bacteria that have entered the VBNC state are not affected in their ability to attach to and invade human erythrocytes, However, incubation with erythrocytes did not resuscitate the VBNC bacteria when plated onto chocolate agar, suggesting that their ability to attach and invade was not due to resuscitation from the VBNC state.

### VBNC *F. tularensis* LVS is insensitive to gentamicin

**FIGURE 5:**
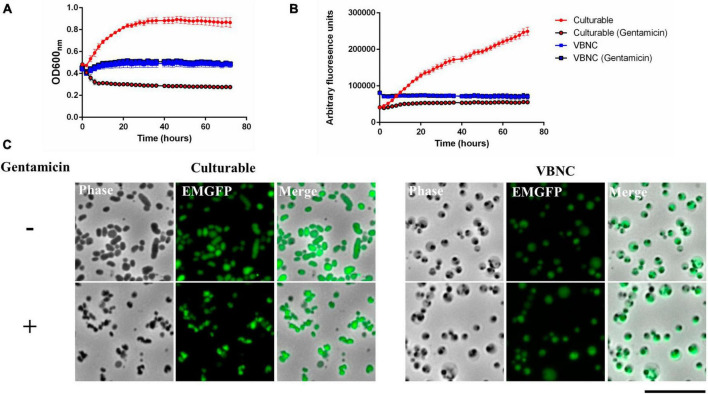
Viable but non-culturable state (VBNC) cells are insensitive to gentamicin. *Francisella tularensis* LVS cells expressing *emgfp* were cultured in CDM for either 24 h (Culturable) or for 504 h (VBNC) at 37°C. Cultures were diluted to a starting OD_600_ of 0.1 and placed in a 96-well plate for incubation with shaking at 37°C with or without 100 μg/ml gentamicin. Optical density **(A)** and fluorescence **(B)** was measured at 2-h intervals for 72 h. Six biological replicates were measured for each strain and error bars show the standard deviation. For culturable cells incubated with gentamicin, optical density fell, and fluorescence remained static. For VBNC cells, both optical density and fluorescence were the same with or without gentamicin. **(C)** *F. tularensis* LVS cells expressing *emgfp* were cultured in CDM for either 24 h (Culturable) or for 552 h (VBNC) at 37°C. Cultures were then incubated for 1 h with (+) or without (-) 100 μg ml^–1^ gentamicin. The culturable bacteria after treatment with gentamicin frequently appeared smaller and irregularly shaped (examples indicated by white arrows) in comparison to non-treated culturable bacteria. The VBNC bacteria did not have an altered appearance after incubation with gentamicin. Scale bar represents 10 μm.

Having determined that VBNC *F. tularensis* LVS can interact with host cells this raised the possibility that bacteria entering the VBNC state during infection may be able to persist in host cells. We were, therefore, interested to see what effect entry into the VBNC state had on susceptibility to antibiotics. Gentamicin is routinely used in cell infection and erythrocyte invasion assays and is an effective antibiotic against *F. tularensis* LVS. Bacteria expressing *emgfp* were grown in CDM for either 24 h (culturable) or 502 h (VBNC), normalized by optical density and diluted into fresh growth medium with or without 100 μg ml^–1^ of gentamicin. Bacteria were incubated at 37°C with shaking and optical density and fluorescence were measured by plate reader at regular intervals. As expected, the culturable bacteria incubated without gentamicin increased in optical density whilst the optical density of the same bacteria incubated with gentamicin decreased and plateaued due to killing. By contrast, there was no change in optical density for the VBNC bacteria, with or without gentamicin (Figure 5A). This suggests that the VBNC bacteria did not die or lyse in the presence of gentamicin. Similarly, when fluorescence was measured, a difference was observed for culturable bacteria, with or without gentamicin, whereas the fluorescence of the VBNC bacteria did not change and was not influenced by incubation with gentamicin (Figure 5B). To confirm that both the drop in density and the loss of fluorescence observed for culturable bacteria was indeed due to killing and lysis because of the gentamicin treatment, culturable and VBNC bacteria were examined microscopically. Distinct changes in morphology could be seen in culturable bacteria incubated with gentamicin (Figure 5C), whereas there was no difference in the microscopic appearance of VBNC cultures with or without gentamicin (Figure 5C). Drip plating of the culturable bacteria exposed to gentamicin for 1 h also confirmed that these cultures were sensitive to gentamicin, producing fewer CFU ml^–1^ than untreated culturable bacteria (data not shown).

### Transcriptomic analysis

**FIGURE 6:**
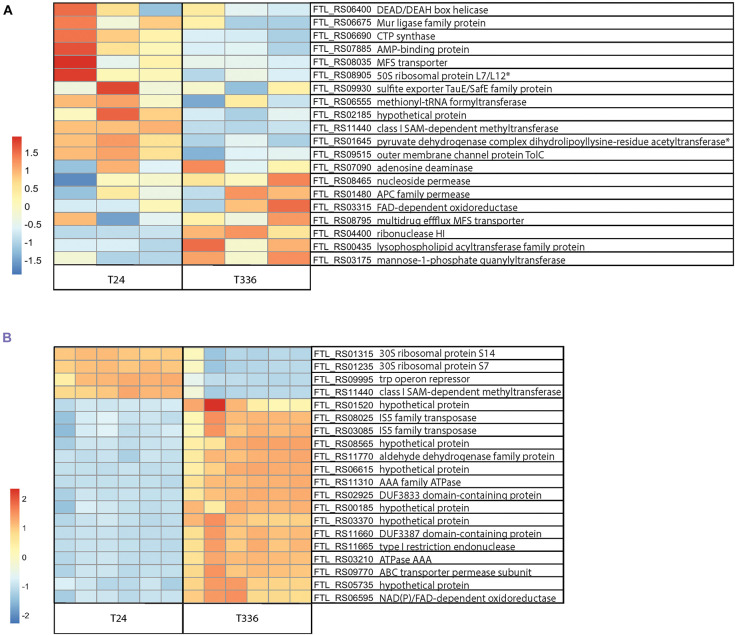
RNA-Seq analysis of *Francisella tularensis* LVS during transition to the VBNC state. *F. tularensis* was cultured in Chamberlain’s Defined Medium (CDM) for 24 and 336 h and RNA isolated from three biological replicates (Nanopore) or six biological replicates (Illumina) was sequenced. **(A)** Top 20 DEG normalized count differences between the T24 and T336 Nanopore experiment samples; associated FTL identifiers and products in table, non-significant DEGs indicated with *. **(B)** Differences in the top 20 most significant DEGs between the T24 and T336 Illumina experiment samples; associated FTL identifiers and products in table.

Very little is known about the molecular mechanisms that promote entry into the VBNC state and, for *F. tularensis* LVS, it is not clear what factors are required for resuscitation. To try to learn more about both the transition into the VBNC state and molecular pathways which may allow resuscitation *in vitro*, we turned to transcriptomics. Initially it was unclear whether such an approach was possible, as VBNC cells may not contain sufficient mRNA for analysis due to lack of transcriptional activity, however, this study shows that quantification of expression is possible even at the most extreme time point evaluated (T336). As expected, the extracted RNA yield dropped notably at the T96 and T336 timepoints for the Nanopore pilot study and at T336 for the samples ultimately sequenced on the Illumina platform, however, there was sufficient RNA for library generation and sequencing in all cases. Approximately 8 million reads passing the Q10 quality filter were generated from the Nanopore sequencing run with an estimated N50 of 272 base pairs. Analysis of the sequences revealed clear differences between the transcriptome of the culturable bacteria at T24 when compared to T336 for VBNC bacteria (Figure 6A). In total there were 145 transcripts that showed a twofold or greater upregulation and 203 genes that were downregulated in VBNC bacteria at T336 (adjusted *p*-value < = 0.01). A total of 496 million reads with at least 10 million reads obtained for each Illumina library, (∼2 million reads per library is recommended for bacterial RNA-Seq) were sequenced on the Illumina platform. Quality scores for over 90% of reads were Q30 or better indicating an inferred base call accuracy of 99.9%. RNA-Seq analysis with DESeq2 showed differences in gene expression between the 24-h (culturable) and 336-h (VBNC) time points (Figure 6B). A preliminary functional analysis of the 32 unique protein IDs found to be expressed in VBNC at the 336 time point in both experiments reveals that gene families involved in transport or metabolism of carbohydrates and amino acids, were upregulated in the VBNC state (Supplementary File 1). Notably, transposases were the predominant locus category detected, representing about 50% of the upregulated *F. tularensis* loci in both experiments at the T336 timepoint. Conversely, 74 unique protein IDs were found to be significantly down regulated in both experiments (Supplementary File 2). Amongst these were genes involved in translation and energy metabolism, which would be consistent with bacteria that had entered a state of lower metabolic activity. The 48, 96, and 240- h time points may represent an intermediate phase during the transition to the full VBNC state and will be explored in future publications. Lists of the most significant (*p* < = 0.1) DEGs detected at a log fold change level >2 in both sequencing experiments along with raw count data can be found in Supplementary Files 3–5, respectively. For comparison, the upregulated and down regulated DEGs found in each experiment and DEGs shared between experiments are shown in Supplementary File 6.

## Discussion

The observed changes in shape and entry into the VBNC state occurred spontaneously in broth cultures of CDM and TSBc (Figure 1). TSBc was used for some of the initial experiments, however, for cell infection and RBC invasion assays CDM gives more reproducible results and so this medium was preferred for subsequent experiments. Broadly, there was no difference in either morphological differentiation or entry into the VBNC state between bacteria cultured in CDM and TSBc. The same phenomena were observed for bacteria cultured in Brain Heart Infusion broth and Mueller Hinton broth (not shown).

For this study we focused on four main time points (24, 48, 96, and 336 h), however, any microscopic observations we made of the VBNC cells cultured for longer than 336 h showed the same cell morphology and plating experiments failed to yield any CFUs. Interestingly, it is possible to resuspend desiccated cultures that have been dried out and maintained under ambient conditions for up to 1 year. These resuspended bacteria retain the heterogenous, coccoid morphology observed after 336 h in broth culture. Staining with FM 4–64 shows that these cells have membranes and some of the cells stain for DNA which suggest that they could be viable (Supplementary Figure 2). However, these cells could not be resuscitated in broth or on chocolate agar plates.

Many bacteria, including *F. tularensis* have been shown to enter the VBNC state under nutrient limiting conditions and in colder temperatures (Xu et al., 1982; Forsman et al., 2000; Ramamurthy et al., 2014; Zhong and Zhao, 2019; Golovliov et al., 2021). For intracellular pathogens, this likely represents a transition from replication in the host to environmental conditions. It has been speculated for *F. tularensis* that the downshift in temperature and nutrients would likely occur as the bacteria transition from a host to an aquatic environment and that entry into the VBNC state would be an adaptation to this (Hennebique et al., 2019). Entry into the VBNC state is common for aquatic and marine bacteria and may be an adaptation to oligotrophic and adverse environments found in nature (Bodor et al., 2020). Our results show that *F. tularensis* LVS enters the VBNC state in broth culture at 37°C and these conditions may more accurately reflect those encountered by the bacteria during replication within a host (Hazlett et al., 2008).

The morphological plasticity of *F. Tularensis* has been noted even in the earliest reports of this bacterium (Hesselbrock and Foshay, 1945). The large, globular cells observed in the VBNC populations reported here are similar to some of those earliest reported morphologies and also have similarities to morphologies reported for mutants of *F. tularensis* that are affected in shape (Robertson et al., 2014; Spidlova et al., 2018; Bachert et al., 2019). The observed change in shape for *F. tularensis* LVS is consistent with what is known from other bacteria which also undergo morphological changes during the entry into the VBNC state (Zhong et al., 2009; Krebs et al., 2011; Pawlowski et al., 2011; Kim et al., 2018). We used FM 4–64, a membrane stain which incorporates into the outer leaflet of membranes and which most efficiently stains growing cells (Haugland, 1996; Pogliano et al., 1999). FM 4–64 staining of culturable *F. tularensis* LVS was very uniform, whereas membrane staining in older cultures was not. A reduction in FM 4–64 staining would be consistent with bacteria entering stationary phase or the VBNC state. However, there were very brightly staining portions of cells in the older cultures (Figure 1A), although it is not clear if this indicates active remodeling of cellular membranes or altered composition, however, changes in membrane and cell envelope composition have previously been reported for VBNC bacteria (Signoretto et al., 2002; Day and Oliver, 2004; Muela et al., 2008; Li et al., 2014). Transmission electron micrographs revealed that, as populations of cells age, the proportion of detached outer membranes increases (Figure 2). This detachment of the outer membrane could be a contributing factor in the observed change in morphology observed in phase contrast micrographs. The outer membrane of *F. tularensis* plays an important role in pathogenesis and stress response and is involved in the production of outer membrane vesicles (OMV’s) (Rowe and Huntley, 2015). OMV’s produced by *Francisella* species can deliver a variety of virulence factors including toxins, effector proteins and outer membrane proteins (OMP’s) (McCaig et al., 2013; Chen et al., 2017; Champion et al., 2018; Sampath et al., 2018; Klimentova et al., 2019) which can vary in response to external conditions (Klimentova et al., 2019). *F. tularensis* also produces a capsule-like-complex, composed of the O-antigen subunit of LPS which can present as an electron-lucent, thin structure surrounding cells in electron micrographs (Hood, 1977; Apicella et al., 2010; Rowe and Huntley, 2015). We did not see evidence for abundant capsule production at any of the time points tested and did not see any outer membrane vesicles in the electron micrographs. We regularly see OMVs in 24-to-48-h samples of *F. tularensis* LVS grown in either TSBc or CDM stained with FM 4–64 (data not shown), so it was surprising that they were not observed in the TEM’s. It is possible that the TEM processing was not conducive to detection of OMV’s and this may also have been the case for capsular material. Future experiments focused on the composition of the outer membrane in VBNC *F. tularensis* LVS would be required to understand the changes observed. For example, changes in outer membrane protein composition may contribute to detachment and the composition of the outer membrane may influence host-microbe interactions. Similarly, it would be interesting to determine the role of peptidoglycan synthesis and composition in the morphological changes observed in VBNC *F. tularensis* LVS as mutants for peptidoglycan synthesis have been shown to have altered morphology (Spidlova et al., 2018; Zellner et al., 2021).

*Francisella tularensis* can infect a wide range of host cells, with phagocytic cells such as macrophages representing the primary route of infection (Celli and Zahrt, 2013). Pathogenic strains of *F. tularensis*, including *F. tularensis* LVS, can evade complement-mediated lysis by altered binding of complement component C3 and conversion of C3b to C3bi, promoting opsonization but not lysis (Clay et al., 2008). After uptake by phagocytic cells, virulent strains of *F. tularensis* inhibit production of proinflammatory cytokines such as TNF-α (Telepnev et al., 2003) and, ultimately, escape destruction by blocking phagolysosomal maturation. Blocking the maturation of the phagolysosome is dependent on the Type Six Secretion System (T6SS) and likely requires effector proteins such as OpiA and PdpC (Lindgren et al., 2004; Uda et al., 2014; Eshraghi et al., 2016; Ledvina et al., 2018; Brodmann et al., 2021). Initial uptake of VBNC *F. tularensis* is not abolished and these bacteria are not efficiently cleared by the macrophages (Figures 3B, C), presumably because complement activation and T6SS activity is not altered, although we cannot rule out the possibility of other mechanisms being involved. However, in contrast to culturable bacteria, VBNC *F. tularensis* LVS provoked a strong induction of TNF-α which was not seen for VBNC cells that were heat-killed (Figure 3A) which might indicate that any factor responsible for the upregulation caused by VBNC bacteria is sensitive to this treatment. Attenuation of TNF-α production has been attributed to the atypical Lipid A of the LPS found in the outer membrane of *F. tularensis*, which evades recognition by Toll-Like Receptor 4 (TLR4) in human cells and by caspase-11 in mouse macrophages (Lagrange et al., 2018; Degabriel et al., 2023). Whilst phase variation of LPS has been described for *F. tularensis* LVS (Soni et al., 2010; Rowe and Huntley, 2015) it is not known if this occurs in VBNC bacteria, or whether this would affect recognition by host cell receptors. The canonical Lipid A biosynthetic pathway from *E. coli* comprises nine enzymes, LpxA, LpxB, LpxC, LpxD, LpxH, LpxK, LpxL, LpxM, and WaaA (Opiyo et al., 2010). *F. tularensis* LVS contains 8 genes predicted to be involved in this pathway, including two homologs of LpxD. The two copies of LpxD in *F. tularensis* have been shown to alter the length and distribution of acyl chains added to LipidA in a temperature dependent manner, with LpxD1 being required for virulence in warm-blooded mammalian hosts (Li et al., 2012). Interestingly, LpxD1 (WP_003014857) is the only gene in the Lipid A biosynthetic pathway that was found to be differentially expressed, being detected in both the Illumina and Oxford Nanopore sequencing experiments as significantly downregulated at 336-h compared to the 24-h samples (Supplementary File 2).

The ultimate fate of VBNC bacteria after uptake is also not known. Microscopy shows that they are phagocytosed but are not cleared by the macrophages and nor do they appear to proliferate within the macrophages under the conditions tested. Some VBNC bacteria, such as *Legionella pneumophila* are known to be able to proliferate in host cells but remain in an unculturable state (Dietersdorfer et al., 2018). Whilst we did not observe any resuscitation from the VBNC state or activity of VBNC cells within the macrophages, our observations raise the possibility that VBNC *F. tularensis* may be able to persist in host cells until activation at a later time by as yet unknown cues.

Perhaps more surprising than the ability to evade clearance in murine macrophages was the observation that VBNC *F. tularensis* LVS cells could be seen invading human erythrocytes (Figure 4). *F. tularensis* is able to invade red blood cells (Horzempa et al., 2011) and, like macrophage infection, this process is dependent on the T6SS (Schmitt et al., 2017). We observed that VBNC bacteria attached to and invaded human erythrocytes at similar frequencies to culturable bacteria, whereas a culturable *iglC*-null mutant was severely affected. We have tried using incubation with erythrocytes to resuscitate VBNC bacteria, but without success, indicating that it is not resuscitation that leads to this phenotype. Human erythrocytes do not possess any phagocytic capacity and the dependence on a functioning TS66 suggests that erythrocyte invasion is an active process. However, the mechanisms by which culturable bacteria can invade red blood cells are not fully understood. The role of the TS66 effector protein, PdpC has been demonstrated (Cantlay et al., 2022) and the erythrocyte cytoskeleton is also required for invasion (Schmitt et al., 2017). Notably, expression of *iglC* and also of *iglB* (which encodes the large subunit of the contractile sheath of the T6SS) were among the subset of genes that were found to be downregulated at the 336-h timepoint in the transcriptomic experiments (Supplementary File 2). Whilst expression of *iglC* was lower in the VBNC bacteria tested, transcripts were still present at detectable levels, indicating that the T6SS system could be active. It is also not known if expression of these genes, or others required for red blood cell invasion, is altered in VBNC cells incubated with erythrocytes. The transcriptional response of VBNC bacteria to potential host cells, such as erythrocytes or macrophages, could be tested in future transcriptional studies.

Due to the reduction in metabolism, VBNC bacteria are generally resistant to antibiotics that target active cellular processes (Oliver, 2010). Our results confirm that VBNC *F. tularensis* LVS also has increased resistance to antibiotics, specifically to gentamicin (Figure 5). It will be important to understand if other factors, such as changes in permeability of the cell envelope contribute to antibiotic resistance. It has been proposed that temperature dependent changes in membrane permeability influences gentamicin sensitivity in *F. tularensis* LVS (Loughman et al., 2016). The physical changes seen in the outer membrane of VBNC *F. tularensis* LVS may also affect permeability for various antibiotics. It will also be interesting to test if antibiotic challenge can promote entry into the VBNC state, as has been observed for *B. subtilis* (Morawska and Kuipers, 2022). Also, whilst we show that there is no change in optical density, fluorescence, or shape in the VBNC cells when challenged with gentamicin, we cannot exclude the possibility that these VBNC bacteria are, in fact being killed, but in contrast to culturable cells do not manifest any changes in morphology or optical density.

Transcriptomics was first applied as a tool to analyze VBNC populations of *Vibrio cholerae* (Asakura et al., 2007), and is increasingly being used to investigate the molecular mechanisms involved in the transition into and maintenance of the VBNC state in bacteria (Liu et al., 2016; Su et al., 2016; Zhong and Zhao, 2019; Morawska and Kuipers, 2022). We used two sequencing platforms, Oxford Nanopore and Illumina to analyze the transcriptional changes in *F. tularensis* LVS as it enters into the VBNC state (Figure 6). In both experiments we saw clear differences in transcription between culturable bacteria grown for 24 h and VBNC bacteria that had been cultured for 2 weeks. As expected, we saw differences in the genes involved in cellular metabolism with evidence that many catabolic pathways are upregulated in the VBNC state. However, we also saw an upregulation of genes encoding putative transporters for amino acids and carbohydrates, suggesting that whilst many biosynthetic pathways are shut down, VBNC bacteria may retain the capacity to acquire nutrients from the environment necessary for maintenance or sense potential signals for resuscitation. Interestingly, our analysis revealed that a high proportion of upregulated transcripts identified at the 336-h (VBNC) time point were sequences associated with transposases. *Francisella* genomes contain a high number of Insertion Sequence (IS) Elements and the transposases encoded in these IS elements have been shown to be highly transcribed (Carlson et al., 2009; Sarva et al., 2016) and may have been involved in the acquisition of virulence traits in the evolutionary history of pathogenic strains of *F. tularensis* (Rohmer et al., 2007). We also detected differential expression of genes predicted to be involved in lipid metabolism and in outer membrane protein production which would be consistent with the morphological changes seen in the VBNC bacteria.

Whilst both sequencing experiments showed that there was differential expression of genes in the VBNC state, there were differences in the sets of genes identified (Supplementary File 6). This is most likely due to differences in the two sequencing methodologies. Nanopore sequencing was preferred for the pilot experiment because of its relatively low cost and accessibility. This experiment proved not only that mRNA transcripts were present in the VBNC bacteria, but also that there were detectable differences in expression compared to culturable bacteria. However, a limitation of the Nanopore platform is that it generated fewer reads and showed much greater variability between the replicates. Because of this, the Nanopore sequencing experiment is likely to have missed differences in genes with relatively low expression and is expected to have a higher rate of false positive and negative results. Nevertheless, this initial experiment gave us the impetus to conduct an experiment using the Illumina platform which generated substantially more sequence reads and sequencing depth and therefore allowed for a more robust differential gene expression analysis. The differentially expressed genes detected by both experiments, and therefore highly likely to be significant for the VBNC state, were of particular interest. Among the differentially expressed genes detected in either experiment were putative transcriptional regulators and future experiments are planned to determine if any of these could be global regulators of the VBNC process in *F. tularensis*.

The transcriptional control of entry into the VBNC state is not well understood, however, there have been studies that show that RpoS, a stress response regulator that coordinates many cellular responses to stress such as metabolism and membrane composition and OxyR, a LysR-family transcriptional regulator have altered expression in VBNC bacteria. RpoS has been shown to influence the VBNC state in *E. coli* (Boaretti et al., 2003) and in *Salmonella enterica* (Kusumoto et al., 2012). Levels of RpoS are influenced by accumulation in cells of (p)ppGpp which is a general indicator of the nutritional status of the cell and intracellular levels of (p)ppGpp are modulated by RelA and SpoT (Atkinson et al., 2011; Pacios et al., 2020). Our transcriptomic analysis of VBNC *F. tularensis* LVS revealed a number of putative transcriptional regulators that are upregulated. One of these, FTL_1193 is a putative LysR-family regulator that has previously been shown to be positively regulated by RelA/SpoT during conditions of oxidative stress in *Francisella tularensis* (Ma et al., 2019). OxyR is thought to be required for the response to reactive oxygen species during the induction of the VBNC state (Cuny et al., 2005; Li et al., 2014). Reactive oxygen species, such as hydrogen peroxide, have been shown to be inducers of the VBNC state and addition of peroxidase degrading compounds such as sodium -pyruvate or catalase can restore culturability (Mizunoe et al., 1999). *F. tularensis* LVS possesses a homolog of *katG* (FTL_1504) which encodes a catalase which, along with OxyR, is required for resistance to hydrogen peroxide and oxidative stress (Honn et al., 2017). The *katG* gene was not identified in our study as being significantly differentially expressed. However, we detected a log fold change in expression for *katG* at the 336-h timepoint of −0.91 and −1.4 for the Oxford Nanopore and Illumina sequencing experiments, respectively. The specific role of oxidative stress in the transition to the VBNC state for *F. tularensis* is not known, however, a role for *katG* in this process would be consistent with what is known from other bacteria.

In conclusion, our results show that *F. tularensis* LVS rapidly and spontaneously enters a VBNC state under standard laboratory broth culture conditions and therefore could make a useful model for the study of this process. The entry into the VBNC state is associated with a morphological differentiation which appears at least in part to be due to outer membrane detachment. This morphological change is terminal, and these cells are remarkably robust and appear resilient to desiccation. We also demonstrate that even after the bacteria have entered the VBNC state they retain the ability to interact with host cells and they are resistant to gentamicin. These things together have important implications for persistence of *F. tularensis* in the environment and perhaps even in host cells. Entry in to the VBNC state may represent an adaptation to changing environmental conditions, whether that be from host to aquatic or soil environments or as the bacteria move between hosts and vectors.

Finally, we present the first transcriptomic analysis of VBNC *F. tularensis* LVS cells. This VBNC specific transcriptome will form the basis for future studies aimed at dissecting the molecular mechanisms that are involved in entry, maintenance and potentially resuscitation from the viable but non-culturable state.

## Data availability statement

The data presented in the study are deposited in the SRA repository under BioProject accession PRJNA1068626 (SRR27715451 to SRR27715480).

## Ethics statement

Ethical approval was not required for the studies on animals in accordance with the local legislation and institutional requirements because only commercially available established cell lines were used.

## Author contributions

SC: Conceptualization, Funding acquisition, Investigation, Methodology, Project administration, Supervision, Writing – original draft, Writing – review and editing. NG: Data curation, Formal Analysis, Software, Visualization, Writing – original draft, Writing – review and editing. RP: Investigation, Writing – review and editing. KW: Investigation, Writing – review and editing. ZK: Investigation, Writing – review and editing. JuF: Investigation, Writing – review and editing. DP: Investigation, Methodology, Writing – review and editing. MS: Investigation, Writing – review and editing. JoF: Investigation, Writing – review and editing. DS: Methodology, Writing – review and editing. JH: Conceptualization, Funding acquisition, Methodology, Writing – review and editing.

## Supplementary material

Supplementary Figure 1 Fluorescence microscopy of *F. tularensis* LVS cultures before and after differentiation shows DNA, membrane and cytoplasmic proteins are maintained. Cultures of *F. tularensis* LVS grown in TSBc at 37°C for either 24 h (T + 1) or 840 h (T + 35). Samples were spotted onto pads of 1% agarose in PBS and visualized using an Olympus IX73 microscope equipped with a 100x NA. 1.30 Phase objective and an Olympus XM10 CCD camera. Scale bars represent 10 μm. **(A)** Membrane. Cells were incubated with 10 μg ml^–1^ of FM-4-64. **(B)** DNA. Cells were incubated with 12 μg ml^–1^ Syto9. **(C)** tdTomato. *F. tularensis* LVS containing the self-replicating plasmid pT3CD expressing *tdtomato*.

Supplementary Figure 2 Fluorescence microscopy of desiccated *F. tularensis* LVS cultures. Cultures of *F. tularensis* LVS grown in CDM at 37°C were allowed to dry out and were kept at room temperature in ambient conditions for 365 days. Cultures were resuspended in PBS for 10 min with gentle shaking at room temperature. Pelleted cells were fixed for 1 h in 2.5% glutaraldehyde in PBS, washed 2 times in PBS and incubated in 10 μg ml^–1^ of FM-4-64 to stain membranes and 10 μg ml^–1^ of Hoechst 33342 to stain DNA. Samples were spotted onto pads of 1% agarose in PBS and imaged with an Olympus IX73 microscope equipped with a 100x NA. 1.30 Phase objective and an Hamamatsu Orca Flash 4.0 LT sCMOS camera. Scale bar represents 10 μm.

Supplementary Figure 3 Comparison of transmission electron micrographs and phase contrast micrographs of VBNC *F. tularensis* LVS. Cells incubated in CDM at 37°C for 336 h were fixed in 2.5% glutaraldehyde at the indicated timepoints and imaged by TEM **(A)** and phase contrast microscopy **(B)**. Scale bars represent 400 nm.

Supplementary Figure 4 Viable But Non-culturable state (VBNC) bacteria can be taken up by RAW 264.7 macrophages but do not proliferate or kill the host cells. A total of 1 × 10^5^ RAW 264.7 cells were seeded in 35 mm dishes to give confluent growth after 24 h. Cells were then incubated with EmGFP labeled *F. tularensis* LVS strains at an MOI of 100 for 24 h. Dishes incubated with culturable *F. tularensis* (grown for 24 h in CDM) had fewer RAW 264.7 cells compared to dishes with either VBNC *F. tularensis* (grown for 720 h in CDM) or an *iglC*-null strain which is defective in intracellular replication. Culturable and VBNC *F. tularensis* localizing inside macrophage cells are indicated by white arrows. The *iglC*-null strain was not observed in RAW 264.7 cells. Scale bar equals 50 μm.
