# Corrigendum: Phenotypic and transcriptional characterization of *F. tularensis* LVS during transition into a viable but non-culturable state

**DOI:** 10.3389/fmicb.2024.1407526

**Published:** 2024-04-22

**Authors:** Stuart Cantlay, Nicole L. Garrison, Rachelle Patterson, Kassey Wagner, Zoei Kirk, Jun Fan, Donald A. Primerano, Mara L. G. Sullivan, Jonathan M. Franks, Donna B. Stolz, Joseph Horzempa

**Affiliations:** ^1^Department of Biomedical Sciences, West Liberty University, West Liberty, WV, United States; ^2^Department of Biomedical Sciences, Joan C. Edwards School of Medicine, Marshall University, Huntington, WV, United States; ^3^Department of Cell Biology, Center for Biologic Imaging, University of Pittsburgh, Pittsburgh, PA, United States

**Keywords:** *Francisella tularensis*, viable but non-culturable (VBNC), RNA-Seq, transcriptomics, bacterial physiology, host-microbe interaction

In the published article, there was an error in the legend for Figure 6 as published. The change in the Figure legend pertains to Figure 6A. The DEGs were identified by normalized count differences and, whilst all but two were also statistically significantly different, the wording has been changed to make it clear that normalized counts were used to identify and rank the genes for the nanopore experiment and the non-significant DEGs are indicated. Also, there was an error in Figure 6 as published. The protein product names in Figure 6 did not match the FTL_RS identifiers due to a sorting error when the names were added in the figure editing process (Excel->Adobe Illustrator). These protein names were manually added using a list of genes for each analysis (nanopore and illumina) that were sorted differently from the clustered order the heatmap figure generated. We have verified that the FTL IDs associated with the heatmaps are accurate in the corrected Figure 6. The protein names have been sorted properly and added to the fixed version of Figure 6. The corrected Figure 6 and its caption appear below.

**Figure 6:**
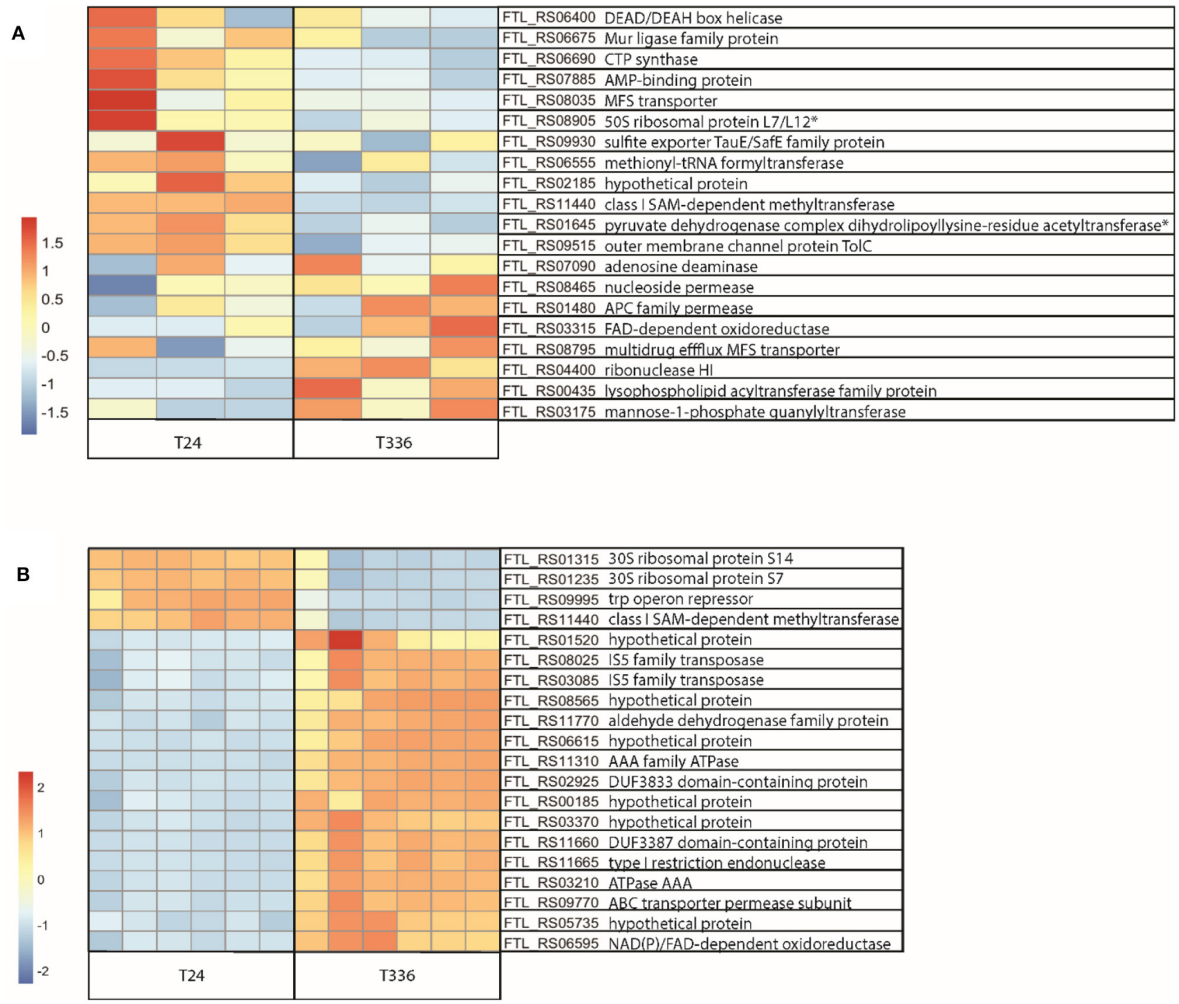
RNA-Seq analysis of *Francisella tularensis* LVS during transition to the VBNC state. *F. tularensis* was cultured in Chamberlain's Defined Medium (CDM) for 24 and 336 h and RNA isolated from three biological replicates (Nanopore) or six biological replicates (Illumina) was sequenced. **(A)** Top 20 DEG normalized count differences between the T24 and T336 Nanopore experiment samples; associated FTL identifiers and products in table, non-significant DEGs indicated with ^*^. **(B)** Differences in the top 20 most significant DEGs between the T24 and T336 Illumina experiment samples; associated FTL identifiers and products in table.

The authors apologize for this error and state that this does not change the scientific conclusions of the article in any way. The original article has been updated.

