# Supplementary figures and images for: Phenotypic and transcriptional characterization of F. tularensis LVS during transition into a viable but non-culturable state
Source: Front Microbiol. 2024 Feb 6;15:1347488. doi: 10.3389/fmicb.2024.1347488 (PMC10877056; doi:10.3389/fmicb.2024.1347488)

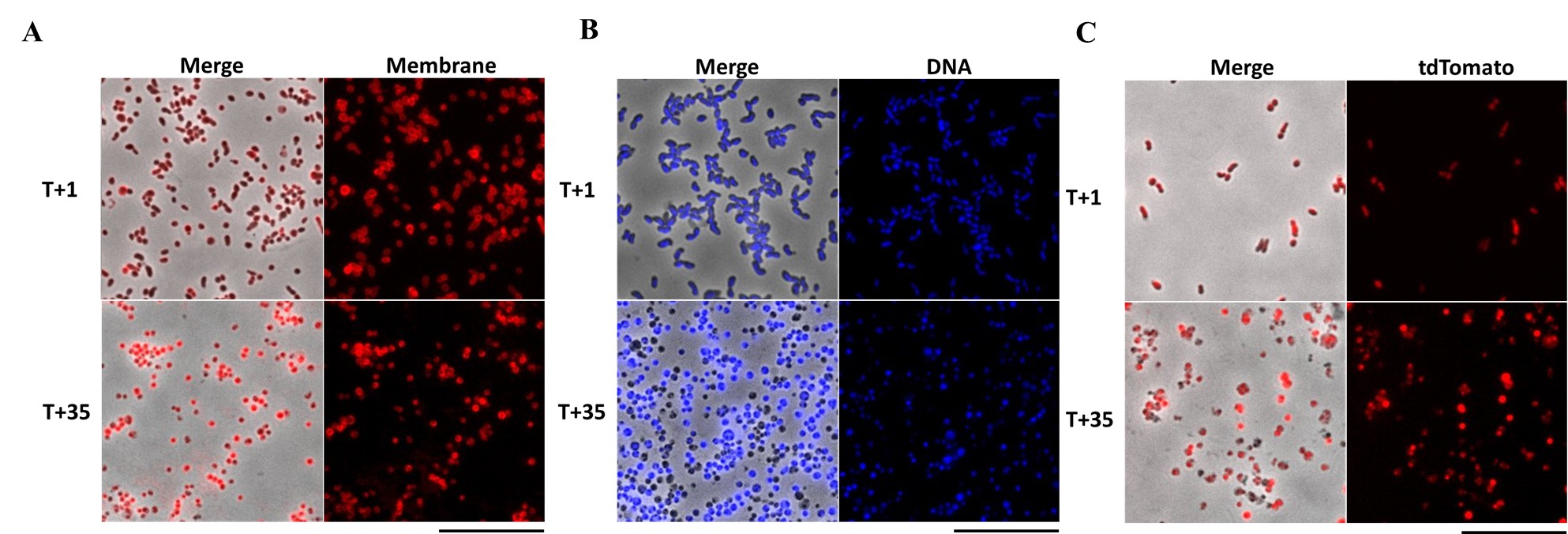

Supplement: Supplementary Figure 1 — Fluorescence microscopy of F. tularensis LVS cultures before and after differentiation shows DNA, membrane and cytoplasmic proteins are maintained. Cultures of F. tularensis LVS grown in TSBc at 37°C for either 24 h (T + 1) or 840 h (T + 35). Samples were spotted onto pads of 1% agarose in PBS and visualized using an Olympus IX73 microscope equipped with a 100x NA. 1.30 Phase objective and an Olympus XM10 CCD camera. Scale bars represent 10 μm. (A) Membrane. Cells were incubated with 10 μg ml–1 of FM-4-64. (B) DNA. Cells were incubated with 12 μg ml–1 Syto9. (C) tdTomato. F. tularensis LVS containing the self-replicating plasmid pT3CD expressing tdtomato. [file Image_1.JPEG]

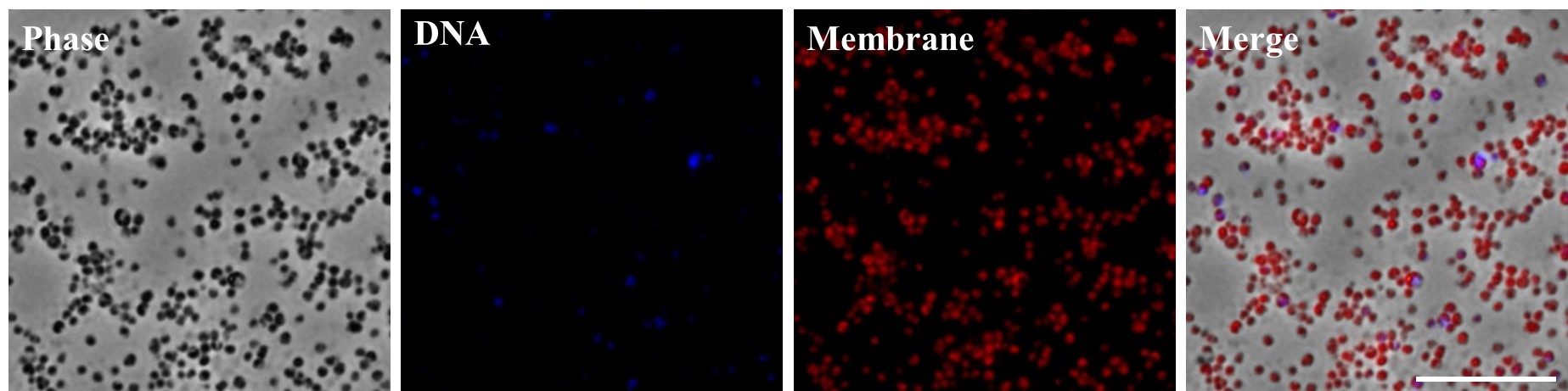

Supplement: Supplementary Figure 2 — Fluorescence microscopy of desiccated F. tularensis LVS cultures. Cultures of F. tularensis LVS grown in CDM at 37°C were allowed to dry out and were kept at room temperature in ambient conditions for 365 days. Cultures were resuspended in PBS for 10 min with gentle shaking at room temperature. Pelleted cells were fixed for 1 h in 2.5% glutaraldehyde in PBS, washed 2 times in PBS and incubated in 10 μg ml–1 of FM-4-64 to stain membranes and 10 μg ml–1 of Hoechst 33342 to stain DNA. Samples were spotted onto pads of 1% agarose in PBS and imaged with an Olympus IX73 microscope equipped with a 100x NA. 1.30 Phase objective and an Hamamatsu Orca Flash 4.0 LT sCMOS camera. Scale bar represents 10 μm. [file Image_2.JPEG]

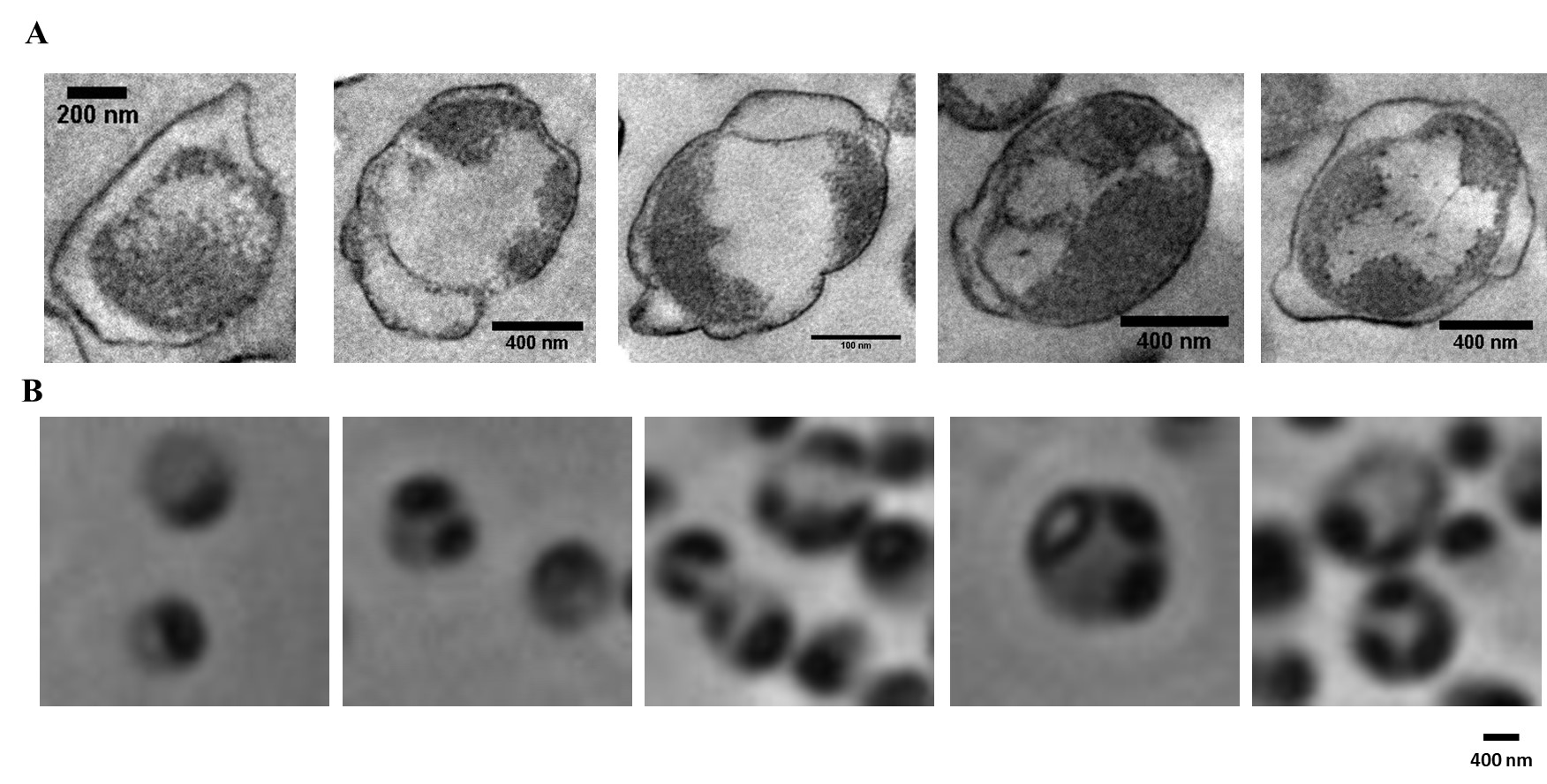

Supplement: Supplementary Figure 3 — Comparison of transmission electron micrographs and phase contrast micrographs of VBNC F. tularensis LVS. Cells incubated in CDM at 37°C for 336 h were fixed in 2.5% glutaraldehyde at the indicated timepoints and imaged by TEM (A) and phase contrast microscopy (B). Scale bars represent 400 nm. [file Image_3.JPEG]

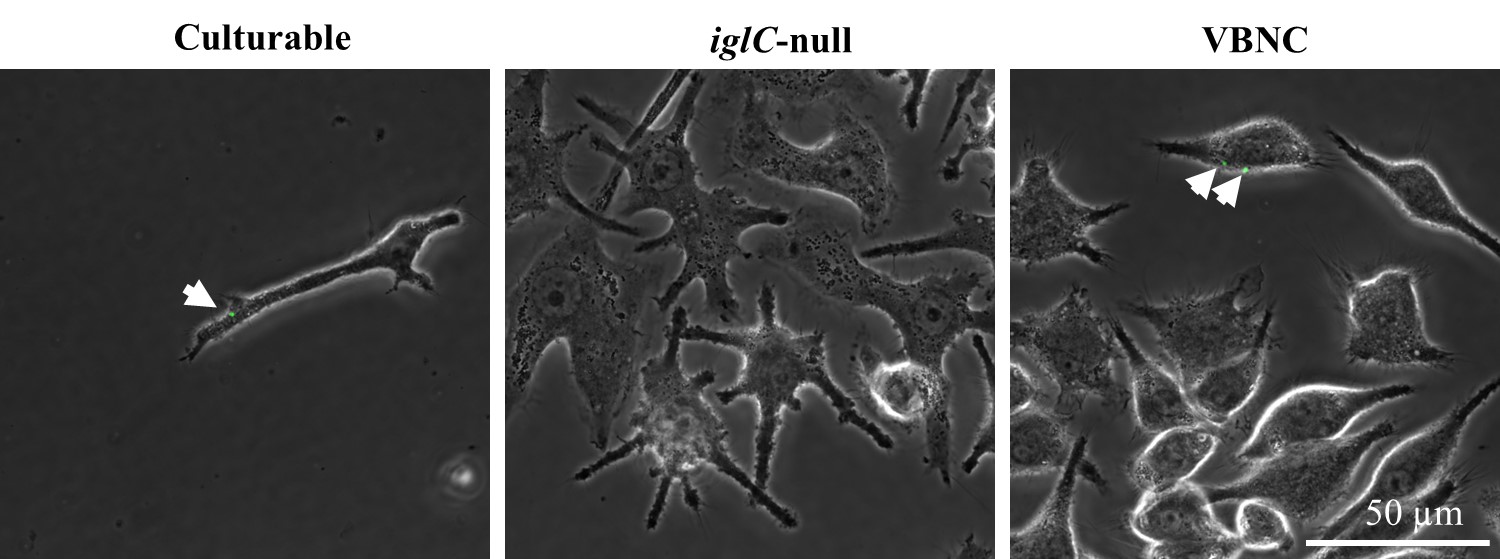

Supplement: Supplementary Figure 4 — Viable But Non-culturable state (VBNC) bacteria can be taken up by RAW 264.7 macrophages but do not proliferate or kill the host cells. A total of 1 × 10^5^ RAW 264.7 cells were seeded in 35 mm dishes to give confluent growth after 24 h. Cells were then incubated with EmGFP labeled F. tularensis LVS strains at an MOI of 100 for 24 h. Dishes incubated with culturable F. tularensis (grown for 24 h in CDM) had fewer RAW 264.7 cells compared to dishes with either VBNC F. tularensis (grown for 720 h in CDM) or an iglC-null strain which is defective in intracellular replication. Culturable and VBNC F. tularensis localizing inside macrophage cells are indicated by white arrows. The iglC-null strain was not observed in RAW 264.7 cells. Scale bar equals 50 μm. [file Image_4.jpg]
