# Supplementary material for: Phenotypic and transcriptional characterization of F. tularensis LVS during transition into a viable but non-culturable state
Source: Front Microbiol. 2024 Feb 6;15:1347488. doi: 10.3389/fmicb.2024.1347488 (PMC10877056; doi:10.3389/fmicb.2024.1347488)
Supplement: Supplementary file 5 [file Table_1.DOCX]

| **RefSeq-Protein** | **Name** | **Old Locus Tag** |
| --- | --- | --- |
| WP_003014006 | Multidrug effflux MFS transporter (Sugar (And other) transporter family protein) | FTL_0734 |
| WP_003014076 | Putative chitinase | FTL_1281 |
| WP_003014144 | Hydrolase | FTL_0063 |
| WP_003014431 | Putative membrane protein | FTL_0093 |
| WP_003014918 | SDR family NAD(P)-dependent oxidoreductase (Short chain dehydrogenase family protein) | FTL_0159 |
| WP_003014922 | Chalcone isomerase domain-containing protein | FTL_0288 |
| WP_003014925 | Mycolic acid cyclopropane synthetase family protein | FTL_0558 |
| WP_003014999 | Phosphoglucomutase/phosphomannomutase, alpha/beta/alpha domain III family protein (Phosphomannomutase) | FTL_0560 |
| WP_003015460 | 3-phosphoshikimate 1-carboxyvinyltransferase (EC 2.5.1.19) (5-enolpyruvylshikimate-3-phosphate synthase) (EPSP synthase) (EPSPS) | FTL_0561 |
| WP_003015463 | Class I SAM-dependent methyltransferase | FTL_0609 |
| WP_003015474 | asparaginase (EC 3.5.1.1) | FTL_0852 |
| WP_003015487 | PIN-like domain-containing protein | FTL_0853 |
| WP_003015496 | Dicarboxylate symporter family protein (Dicarboxylate/amino acid:cation symporter) | FTL_0855 |
| WP_003015502 | MFS transporter, sugar porter family protein (Sugar porter family MFS transporter) | FTL_0860 |
| WP_003016175 | Uncharacterized protein | FTL_0863 |
| WP_003016192 | DUF2345 domain-containing protein | FTL_0865 |
| WP_003016246 | Sodium:solute symporter | FTL_1183 |
| WP_003016271 | Quercetin 2,3-dioxygenase C-terminal cupin domain-containing protein | FTL_1194 |
| WP_003016320 | Amino acid permease (Amino acid permease family protein) | FTL_1220 |
| WP_003016424 | Sugar MFS transporter | FTL_1278 |
| WP_003016434 | Glycoside hydrolase family 3 protein | FTL_1282 |
| WP_003016520 | CRISPR-associated endonuclease Cas1 | FTL_1323 |
| WP_003016595 | FUSC family protein (Fusaric acid resistance family protein) | FTL_1367 |
| WP_003017422 | Lipoprotein | FTL_1731 |
| WP_010030467 | Sel1 repeat family protein | FTL_1853 |
| WP_010032171 | Helix-turn-helix domain-containing protein (Helix-turn-helix family protein) | FTL_0557 |
| WP_011457396 | Sugar (And other) transporter family protein | FTL_0502 |
| WP_011648612 | DUF1365 domain-containing protein | FTL_1472 |
| WP_013921936 | ABC transporter, permease protein | many |
| WP_011457329 | IS630 family transposase | many |
| WP_003019836 | Putative transposase | many |
| WP_003019836 | Transposase | many |
