# Supplementary material for: Phenotypic and transcriptional characterization of F. tularensis LVS during transition into a viable but non-culturable state
Source: Front Microbiol. 2024 Feb 6;15:1347488. doi: 10.3389/fmicb.2024.1347488 (PMC10877056; doi:10.3389/fmicb.2024.1347488)
Supplement: Supplementary file 6 [file Table_2.DOCX]

| **RefSeq-Protein** | **Name** | **Old Locus Tag** |
| --- | --- | --- |
| WP_003013591 | chromosomal replication initiator protein DnaA | FTL_0001 |
| WP_011457328 | OmpH family outer membrane protein | FTL_0009 |
| WP_003013699 | rhodanese-like domain-containing protein | FTL_0010 |
| WP_003014087 | DUF3568 domain-containing protein | FTL_0105 |
| WP_010031646 | type VI secretion system contractile sheath large subunit | FTL_0112 |
| WP_003016165 | type VI secretion system tube protein IglC | FTL_0113 |
| WP_003014289 | 30S ribosomal protein S2 | FTL_0224 |
| WP_003017693 | translation elongation factor Ts | FTL_0225 |
| WP_003014302 | ribosome recycling factor | FTL_0227 |
| WP_003014323 | 30S ribosomal protein S12 | FTL_0232 |
| WP_003021606 | 30S ribosomal protein S7 | FTL_0233 |
| WP_003027202 | 50S ribosomal protein L3 | FTL_0236 |
| WP_003027200 | 50S ribosomal protein L23 | FTL_0238 |
| WP_011457354 | 50S ribosomal protein L2 | FTL_0239 |
| WP_003027195 | 30S ribosomal protein S19 | FTL_0240 |
| WP_010030778 | 50S ribosomal protein L16 | FTL_0243 |
| WP_003014341 | 50S ribosomal protein L29 | FTL_0244 |
| WP_003014349 | 50S ribosomal protein L24 | FTL_0247 |
| WP_003014352 | 50S ribosomal protein L5 | FTL_0248 |
| WP_003014361 | 30S ribosomal protein S5 | FTL_0253 |
| WP_003014378 | 30S ribosomal protein S4 | FTL_0260 |
| WP_003016509 | glycosyltransferase | FTL_0265 |
| WP_003018337 | acetyl-CoA carboxylase carboxyltransferase subunit alpha | FTL_0295 |
| WP_010031777 | pyruvate dehydrogenase (acetyl-transferring)%2C homodimeric type | FTL_0309 |
| WP_003014822 | 50S ribosomal protein L28 | FTL_0522 |
| WP_003014857 | UDP-3-O-(3-hydroxymyristoyl)glucosamine N-acyltransferase LpxD1 | FTL_0537 |
| WP_010030504 | NADP-dependent isocitrate dehydrogenase | FTL_0588 |
| WP_003014965 | Wzz/FepE/Etk N-terminal domain-containing protein | FTL_0589 |
| WP_003015055 | DUF3568 family protein | FTL_0645 |
| WP_003015178 | serine hydroxymethyltransferase | FTL_0703 |
| WP_003015181 | ribosome-associated protein | FTL_0704 |
| WP_003015359 | succinate dehydrogenase assembly factor 2 | FTL_0804 |
| WP_003015669 | low molecular weight phosphotyrosine protein phosphatase | FTL_0905 |
| WP_003015695 | ferritin | FTL_0926 |
| WP_003015722 | isochorismatase family protein | FTL_0941 |
| WP_003015781 | tyrosine--tRNA ligase | FTL_0968 |
| WP_003015877 | peroxiredoxin | FTL_1015 |
| WP_003015897 | 30S ribosomal protein S6 | FTL_1024 |
| WP_003015961 | GatB/YqeY domain-containing protein | FTL_1048 |
| WP_003019053 | FKBP-type peptidyl-prolyl cis-trans isomerase N-terminal domain-containing protein | FTL_1097 |
| WP_003025404 | acyl carrier protein | FTL_1138 |
| WP_010031646 | type VI secretion system contractile sheath large subunit | FTL_1158 |
| WP_003016165 | type VI secretion system tube protein IglC | FTL_1159 |
| WP_003016509 | glycosyltransferase | FTL_1317 |
| WP_011457482 | outer membrane protein FopA | FTL_1328 |
| WP_015083947 | translation initiation factor IF-3 | FTL_1406 |
| WP_003016677 | threonine--tRNA ligase | FTL_1407 |
| WP_003016758 | purine-nucleoside phosphorylase | FTL_1461 |
| WP_003016829 | glycerophosphodiester phosphodiesterase | FTL_1511 |
| WP_003016861 | phosphopyruvate hydratase | FTL_1527 |
| WP_003016895 | bifunctional DNA-formamidopyrimidine glycosylase/DNA-(apurinic or apyrimidinic site) lyase | FTL_1543 |
| NA | pseudogene: similar to DUF2147 domain-containing protein | NA |
| WP_003016972 | type II 3-dehydroquinate dehydratase | FTL_1593 |
| WP_003016993 | DNA polymerase III subunit delta' | FTL_1604 |
| WP_003019455 | glutathione binding-like protein | FTL_1606 |
| WP_011457525 | DNA-directed RNA polymerase subunit beta | FTL_1744 |
| WP_015083957 | 50S ribosomal protein L1 | FTL_1747 |
| WP_003017320 | succinate dehydrogenase flavoprotein subunit | FTL_1786 |
| WP_003019781 | succinate dehydrogenase%2C cytochrome b556 subunit | FTL_1788 |
| WP_003017341 | F0F1 ATP synthase subunit delta | FTL_1798 |
| WP_003017342 | F0F1 ATP synthase subunit B | FTL_1799 |
| WP_010032491 | transcription termination factor NusA | FTL_1810 |
| WP_003024580 | ribosome maturation factor RimP | FTL_1811 |
| WP_003017384 | NADH-quinone oxidoreductase subunit D | FTL_1827 |
| WP_011457538 | NADH-quinone oxidoreductase subunit C | FTL_1828 |
| WP_003017394 | NADH-quinone oxidoreductase subunit B | FTL_1829 |
| WP_003017416 | hypothetical protein | FTL_1849 |
| WP_003017431 | protein-L-isoaspartate O-methyltransferase | FTL_1866 |
| WP_003016509 | glycosyltransferase | FTL_1892 |
| WP_011648794 | 30S ribosomal protein S1 | FTL_1912 |
| WP_003013806 | hypothetical protein | FTL_1914 |
| WP_003013838 | aminodeoxychorismate/anthranilate synthase component II | FTL_1965 |
| WP_010031554 | anthranilate synthase component 1 | FTL_1966 |
| WP_003017545 | trp operon repressor | FTL_1967 |
